# Breaking (Fake) News: No Personal Relevance Effect on Misinformation Vulnerability

**DOI:** 10.3390/bs13110896

**Published:** 2023-10-30

**Authors:** Francesco Ceccarini, Pasquale Capuozzo, Ilaria Colpizzi, Corrado Caudek

**Affiliations:** 1 Department of Science, New York University Abu Dhabi, Abu Dhabi 129188, United Arab Emirates; fc2284@nyu.edu; 2 IRCCS Istituto Giannina Gaslini, 16147 Genoa, Italy; pasqualecapuozzo@gaslini.org; 3 Neurosciences, Psychology, Drug Research and Child Health (NEUROFARBA), University of Florence, 50139 Florence, Italy; ilaria.colpizzi@unifi.it

**Keywords:** misinformation, vulnerability factors, COVID-19, fake news, social media

## Abstract

The massive spread of fake news (FN) requires a better understanding of both risks and protective psychological factors underlying vulnerability to misinformation. Prior studies have mostly dealt with news that do not bear any direct personal relevance to participants. Here, we ask whether high-stakes news topics may decrease vulnerability to FN. Data were collected during the national lockdown in Italy (COVID-19 news) and one year later (political news). We compared truth discrimination and overall belief for true news (TN) and FN concerning COVID-19 and political topics. Our findings indicate that psychological risk and protective factors have similar effects on truth discrimination, regardless of whether the news topic is highly or minimally personally relevant. However, we found different effects of psychological factors on overall belief, for high and low personal relevance. These results suggest that, given a high level of cognitive dissonance, individuals tend to rely on proximal or emotional sources of information. In summary, our study underscores the importance of understanding the psychological factors that contribute to vulnerability to misinformation, particularly in high-stakes news contexts.

## 1. Introduction

Fake news (FN; i.e., news articles that are intentionally and verifiably false) is a significant problem in today’s society, fueled by the proliferation of social media and the ease of sharing information online. The negative impacts of fake news, as well as its potential to influence personal and social life, have promoted extensive research to face this issue.

Two main approaches have been proposed to contrast the spreads of FN: (1) the development of artificial intelligence (AI) algorithms [1], and (2) the utilization of expert knowledge, such as fact-checking organizations [2]. Both methods are able to detect and mitigate the impact of FN, but they also have many limitations: AI algorithms are susceptible to false positives (e.g., unjustified censorship), whereas fact-checking processes are expensive and suffer from scalability and efficiency issues [3].

For these reasons, an alternative route for trying to limit the impact of FN is the study of psychological vulnerability to misinformation. Researchers are trying to figure out why certain individuals are more susceptible to misinformation than others, and which individual traits are associated with a greater vulnerability to FN [4]. This latter line of research has identified many determinants that may increase the likelihood of being misled by FN, including, for example, partisan bias (i.e., the tendency to interpret new information in accordance with one’s ideological beliefs [5]), delusion-proneness [6], dogmatism and religious fundamentalism [6], over-claiming [7], and cognitive reflection (i.e., the disposition to rely on intuitive rather than analytical thinking [8]).

It is important to note, however, that previous studies have mainly focused on political news [9], that is, on a category of news for which misinformation only has limited immediate consequences for the individual. One major concern, therefore, is the generalizability of previous findings to real-life situations, that is, to situations in which being misled by FN produces immediate unfavorable consequences for the individual [10]. In other words, previous studies have scarcely explored the personal relevance [11] of the news topics, which is a fundamental aspect of the individuals’ vulnerability to FN [4]. Indeed, higher personal relevance has been associated with an augmented attention to information, and it is supposed to result in a more significant allocation of cognitive resources toward the processing of the information [12]. In this sense, high personal relevance, by mobilizing more cognitive resources, may reduce susceptibility to FN. However, other studies have challenged this hypothesis. For instance, when exposed to information that aligns with their pre-existing views or beliefs, individuals with high involvement exhibit a higher acceptance rate of such information [13]. In a similar vein, misinformation that holds high personal relevance or involvement proves more challenging to correct compared to misinformation with low involvement [14]. Other studies showed that individuals tend to be motivated to believe in misinformation that fosters a feeling of hopefulness, especially in uncertain and difficult situations [15]. Therefore, it is yet unclear how and to what extent personal relevance of news topics affects an individual’s ability to detect FN.

To address this issue, we evaluated how the personal relevance of news topics affects the ability to distinguish between FN and true news (TN). Specifically, we aimed to explore (1) the determinants of misinformation, and (2) the protective and vulnerability factors making individuals more or less adept at discerning TN and FN. For this purpose, we capitalized on the unique scenario provided by the COVID-19 pandemic [16,17]. The pandemic, a unique global event, provided an exceptional opportunity to naturally imbue related news topics with a high degree of personal relevance, far surpassing what could be artificially obtained in controlled laboratory settings.

In light of this, we examined truth discernment across two sets of news: COVID-19 news (with high personal relevance) and political news (with low personal relevance). Participants were asked to judge the credibility of a set of COVID-19 TN and FN. News concerning the behavioral rules to follow in order to limit the spread of the virus (e.g., washing hands frequently, avoiding touching both nose and mouth, using face masks in public) were (and still are) literally a matter of life or death. Acting in violation to TN (and in accord to FN) in relation to such behaviors increased the probability of self-defeating and irresponsible behaviors [18]. A second data collection took place one year later. Participants were asked to judge the credibility of political TN and FN. In the second scenario the personal relevance of the news topics was much smaller than in the first administration.

## 2. Methods

### 2.1. Participants

The COVID-19 news and the political news samples comprised 651 and 460 participants, respectively (for details, see Supplementary Material).

### 2.2. Material

Prior studies highlighted several psychological traits enhancing the vulnerability to FN. For all the participants, we measured the following psychometric scales: *Fake News Susceptibility Scale* (FNSS; [19]), *Right-Wing Authoritarianism Scale* (RWAS [20]), *Magical Ideation Scale* (MIS [21]), and *Religious and Fundamentalism Scale* (RFS; [22])—see Supplementary Material for details.

We collected two sets of headlines (COVID-19 news and political news) adhering to stringent guidelines for FN corpora construction [23], with an equal distribution of FN and TN within each set. To assess the veridicality of headlines, we relied on well-known fact-checking websites with excellent reliability—see Supplementary Material.

### 2.3. Procedure

The participants were instructed to evaluate 30 headlines (15 TN, 15 FN). Following this, they completed four self-report questionnaires—see Supplementary Material for further details.

### 2.4. Data Analysis

Belief in FN was assessed using two common measures: truth discernment and overall belief [4]. Truth discernment was computed as the degree of belief in TN minus the degree of belief in FN. Overall belief was computed as the sum of the degrees of belief in TN and FN, without distinguishing between them (see Supplementary Material for a comparison with the signal detection theory indices).

For our analyses, we followed a Bayesian approach by fitting linear regression models in R using Stan and the brms package [24]. The question of whether vulnerability and protective psychological factors influence truth discernment and overall belief when manipulating the degree of personal relevance of the news topic was framed as the statistical problem of determining whether the optimal subset of the predictors that can maximally predict an outcome measure in a training sample can generalize to a new out-of-sample data set. We focused on model generalizability because of the increasingly important focus on replicable research. We externally validated our models after variable selection because model complexity affects generalizability: In general, as the model becomes less complex, it better generalizes to other samples in the population.

We reasoned as follows: If the best subset of predictors selected from the political news data set does not reliably decrease predictive accuracy when the regression model is applied to the COVID-19 data set, then we can conclude that the vulnerability and protective factors influence belief in FN (truth discernment and overall belief) in a similar manner for both high and low personal relevance news topics.

The models’ out-of-sample predictive accuracy was assessed using the LOO Bayesian estimate of the expected log pointwise predictive density (ELPD-LOO) and the Bayesian *R*^2^. We made inferences about predictors’ effects based on 95% credible intervals—see Supplementary Material.

External validation was performed by using the political news data to fit a reference model and the COVID-19 news data to validate the models’ predictions.

1. For the reference model based on the political news data, we fitted a Bayesian multiple regression model to predict either truth discernment or overall belief from 14 candidate predictors.
2. After fitting the reference model, we used predictive projection to find the smallest possible submodel that would predict belief in FN (truth discernment or overall belief) almost as well as the reference model.
3. The submodel selected from the political news data was then validated by using the COVID-19 news data. We considered three different scenarios [25].

- *Scenario 1* (the null scenario). The model is completely accurate, that is, both the model structure M (i.e., the specification of the predictors) and the parameter set *θ*_0_ are accurate. In other words, for predicting truth discernment or overall belief in the COVID-19 news data, the model uses the same subset of predictors and the same coefficients that were estimated from the political news data.
- *Scenario 2* (faulty prior information on the model’s parameters Θ). The model structure M is accurate, but information on one or more of the parameters is erroneous. In other words, for predicting truth discernment or overall belief in the COVID-19 news data, the model uses the same subset of predictors that were selected from the political news data, but different coefficients.
- *Scenario 3* (faulty model structure). The model structure itself is wrong, which means that the best subset of predictors that were selected from the political news data does not correspond to the best subset of predictors that can be selected from the COVID-19 news data.

The models’ performance was evaluated according to the criteria described in the Supplementary Material.

## 3. Results

### 3.1. External Validation for News Truth Discernment

**Figure 1:**
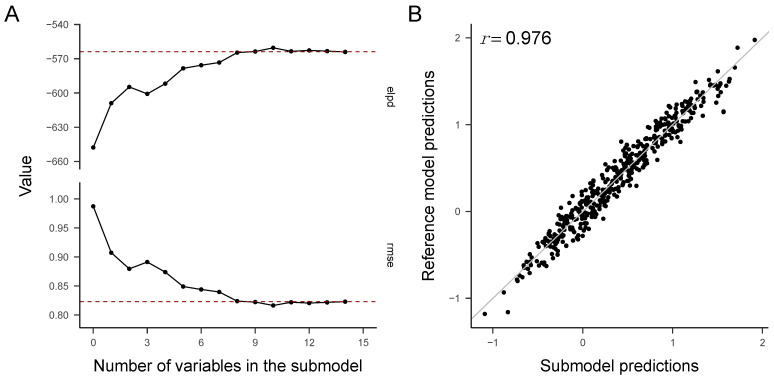
Truth discernment about political news data. Trajectories of the predictive projection feature selection and scatterplot of the predictions of the reference model versus the predictions of the optimal submodel. (**A**) Change in ELPD/decrease in RMSE as more predictors entered the submodel. (**B**) Truth discernment in political news judgment predicted by the reference model (14 predictors) as a function of the truth discernment predicted by the submodel (8 predictors).

**Figure 2:**
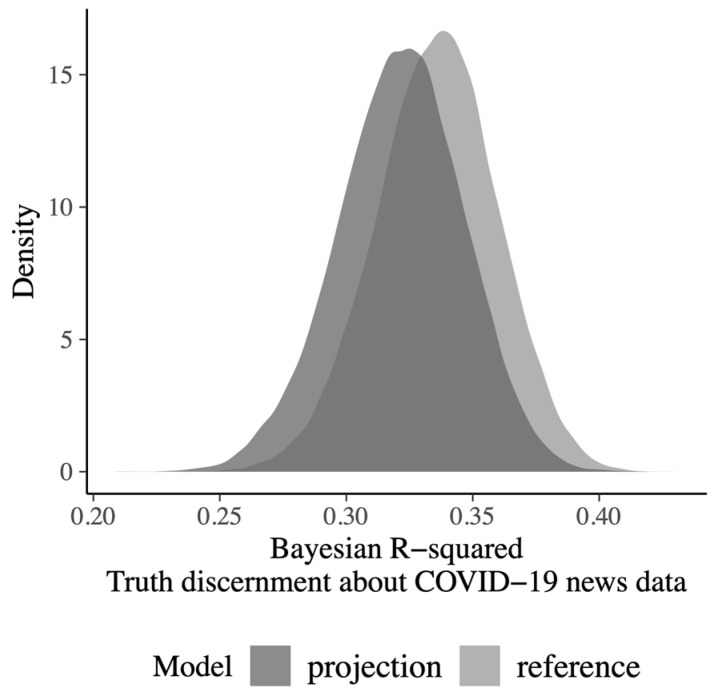
Truth discernment about COVID-19 news data. Bayesian R-squared for the reference model (with 14 predictors) and for the projection model comprising the 8 predictors that were selected from the political news data.

1. For the political news data, we fitted a Bayesian multiple regression reference model to predict news truth discernment from 14 candidate predictors. For this model, we obtained a Bayesian *R*^2^ = 0.35, 95% CI [0.29, 0.41] and a model standard deviation = 0.81, 95% CI [0.76, 0.87], which indicate a moderately good predictive performance—see Supplementary Material for details.
2. By using predictive projection (i.e., by decreasing the Kullback–Leibler divergence from the reference model to the projected submodel), we found that a submodel which included only eight covariates produced a predictive performance similar (according to the 1 SE rule) to that of the reference model with all 14 covariates (Figure 1A). The 1 SE-submodel had a Bayesian *R*^2^ of 0.33, 95% CI [0.27, 0.39] and a model standard deviation of 0.81, 95% CI [0.76, 0.87]. The predicted values of the 1 SE-submodel were strongly associated with the predicted values of the reference model (Pearson *r* = 0.98; see Figure 1B). The eight predictors in the optimal submodel, in the order in which they were entered into the submodel, were age, gender, education, political orientation, RWAS conventionalism, FNSS conspiratorial beliefs, FNSS critical news consumption, and MIS.
3. The crucial point of the statistical analysis was to evaluate the predictive performance of the selected submodel by external validation. The eight covariates selected from the political news data were used as predictors for truth discernment in the COVID-19 news data set. According to Scenario 1 (accuracy of the model structure and of the parameter set), external validation failed: the expected log-predictive density leave-one-out cross-validation difference (Δ ELPD LOO) worsened when it was computed for the COVID-19 news relative to when it was computed for the political news data, Δ ELPD LOO = 994.8 ± 25.7 *SE*. Instead, according to Scenario 2 (accuracy of model structure but not of parameter set), we found that the predictive performance of the selected submodel generalized well to the new data set. For the COVID-19 news data, we found no evidence of a decrease in predictive accuracy when using the eight predictors chosen from the political news data rather than the whole set of 14 predictors in the sample, Δ ELPD LOO = −0.02 ± 3.34 *SE*. For the projected model (eight predictors selected from the political news data), the Bayesian *R*^2^ = 0.32, 95% CI [0.27, 0.37] and the model standard deviation = 0.75, 95% CI [0.71, 0.79] (see Figure 2). When using all the 14 predictors on the COVID-19 news data, the Bayesian *R*^2^ = 0.34, 95% CI [0.29, 0.38] and the model standard deviation = 0.75, 95% CI [0.71, 0.79]. Therefore, for the COVID-19 news data, the performance measures (Δ ELPD LOO, Bayesian *R*^2^) of the projected model (predictors chosen from the political news data) are well-within the uncertainty bounds of the complete model (14 predictors; see Supplementary Material for details).

**Figure 3:**
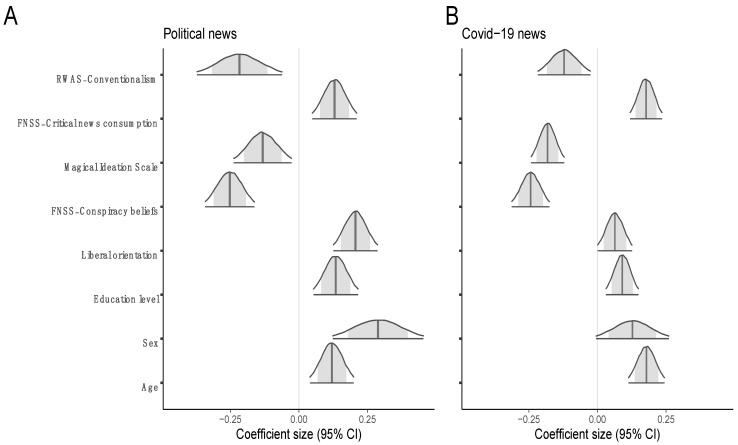
(**A**) Credible intervals for predictors in the submodel for the political news data set. (**B**) Credible intervals for predictors in the submodel for the COVID-19 data set generated by using the same predictors that were selected in the training set (political news data). Inner shaded area = 80% CI.

Figure 3 shows the credible intervals for the eight predictors in the selected submodel that were estimated in the political news data set (panel A) and in the COVID-19 news data set (panel B). Although external validation according to Scenario 1 failed, the pattern of the results is very similar in the two cases. We interpret this as indicating that truth discernment is affected by psychological protective and vulnerability factors in a similar manner when the news has a direct personal relevance (COVID-19 news) and when it has no immediate personal relevance (political news). The 95% CIs always overlap between the two sets of predictors, apart from liberal orientation. Liberal orientation showed a stronger facilitating effect on truth discernment for the political news data, 95% CI [0.13, 0.29], than for the COVID-19 news data, 95% CI [0.001, 0.13]. We do not deem this difference to be surprising, because opinions about COVID-19 were not strongly politically polarized in Italy at the time of the national lockdown—differently from what happened, for example, in the United States, or also in Italy after the national lockdown.

### 3.2. External Validation for Overall News Belief

**Figure 4:**
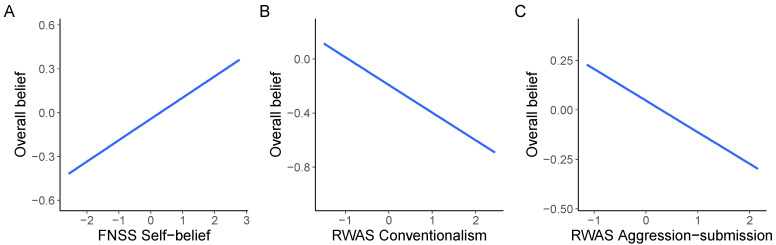
COVID-19 data. Conditional effects of the optimal submodel for predicting overall belief. The three predictors were selected from the original set of 14 predictors by ignoring the political news data. Overall belief tends to increase with (**A**) FNSS self-belief, and it tends to decrease with (**B**) RWAS conventionalism and with (**C**) RWAS aggression–submission.

**Figure 5:**
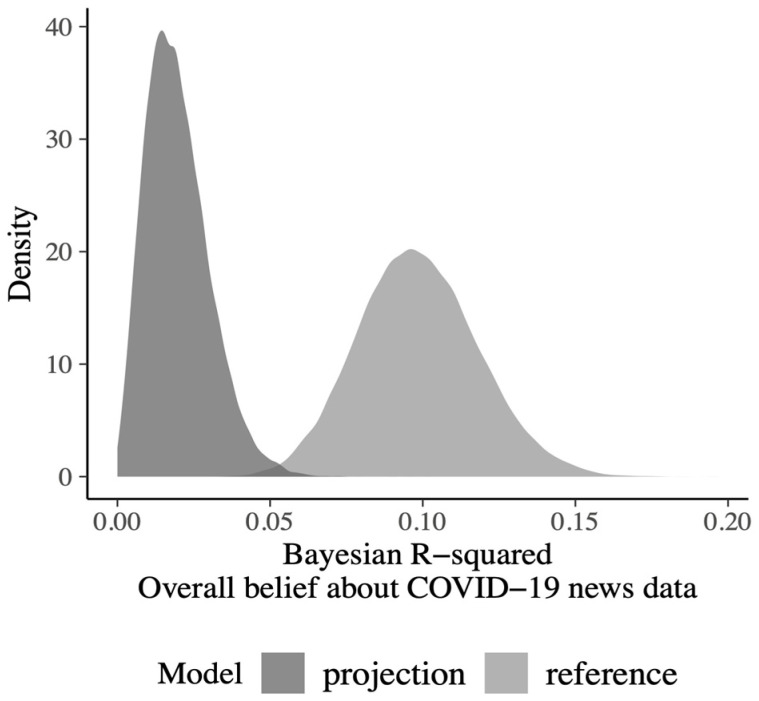
Overall belief about COVID-19 news data. Bayesian R-squared for the reference model (with 14 predictors) and for the projection model comprising one predictor that was selected from the political news data.

1. The Bayesian regression model with 14 covariates (see above) provided the reference model for the political news data; Bayesian *R*^2^ = 0.11, 95% CI [0.06, 0.16], model standard deviation = 1.01, 95% CI [0.95, 1.08]. The 95% CI did not cross the zero for four covariates: age, *β* = 0.10, 95% CI [0.00, 0.21]; education, *β* = 0.14, 95% CI [0.04, 0.24]; MIS, *β* = 0.15, 95% CI [0.01, 0.29]; and FNSS self-belief, *β* = 0.23, 95% CI [0.13, 0.32].
2. By using a forward stepwise addition procedure, we identified the minimal subset of these covariates that had similar predictive power as the reference model. For this submodel, the Bayesian *R*^2^ = 0.05, 95% CI [0.01, 0.08] and the model standard deviation = 1.02, 95% CI [0.96, 1.09]. The only covariate included in the projected model was FNSS self-belief, *β* = 0.22, 95% CI [0.13, 0.32]—see Figure 4.
3. For the COVID-19 news data, however, the optimal submodel included three predictors (FNSS self-belief, *β* = 0.15, 95% CI [0.07, 0.22]; RWAS conventionalism, *β* = −0.20, 95% CI [−0.32, −0.09]; and RWAS aggression–submission, *β* = −0.16, 95% CI [−0.26, −0.06]); with an *R*^2^ of 0.05, 95% CI [0.02, 0.08] and a model standard deviation = 0.95, 95% CI [0.90, 1.00]. For comparison, the full model with 14 covariates had a Bayesian *R*^2^ of 0.10, 95% CI [0.06, 0.14] and a model standard deviation of 0.94, 95% CI [0.89, 0.99], whereas the model with FNSS self-belief as the only covariate (*β* = 0.13, 95% CI [0.06, 0.20]) had a Bayesian *R*^2^ = 0.02, 95% CI [0.00, 0.04] and a model standard deviation of 0.96, 95% CI [0.91, 1.01]—see Figure 5.

In conclusion, we found no evidence of external validation for the overall belief measure (Scenario 3: faulty model structure): The best subset of predictors that can be selected from the political data news does not correspond to the optimal subset of predictors that can be selected from the COVID-19 news data. This indicates that response bias (i.e., overall belief) is affected in a different manner by protective and vulnerability psychological factors for news having a high or a low personal relevance (for details, see Supplementary Material).

## 4. General Discussion

The present study aims to evaluate whether the personal relevance of news topics impacts an individual’s ability to assess FN. For this purpose, we conducted a sort of “natural experiment” in which the range of variation of the independent variable (i.e., the degree of personal relevance of news topics) was incomparably larger than what is achievable through any laboratory manipulation. In these conditions, we found no evidence that the personal relevance of the news topics (high: COVID-19 news; low: political news) can moderate the impact of psychological vulnerability and protective factors on news truth discernment, for the psychological dimensions presently examined.

When considering response bias instead, the present results indicate that overall belief is affected by a different set of predictors, depending on the personal relevance of news topics. The optimal submodel for the political news included only one predictor, FNSS self-belief. The optimal submodel for the COVID-19 news included three predictors: FNSS self-belief, RWAS conventionalism, and RWAS aggression–submission.

Together, the present results indicate that, although it affects the general attitude toward the news (overall belief), the personal relevance of news topics does not affect the ability to discern true from false news.

### 4.1. Determinants of Misinformation

The study of the psychological characteristics that make individuals vulnerable to being misinformed has focused on individual differences in analytical thinking [8], delusion-proneness [6], religiosity and dogmatism [6], bullshit receptivity [26], over-claiming one’s own knowledge [7], confirmation bias [2], selective exposure [27], partisan bias [28], and others. If we consider that all these variables can be operationalized in many different ways, it is certain that external validation according to Scenarios 1 and 2 will fail (see Section 2) if a large set of variables is considered in the statistical analysis. Notwithstanding this necessary fact, the present results are noteworthy because they show that an important subset of predictors of FN vulnerability acts in a very similar manner for news topics with low and high personal relevance.

### 4.2. Vulnerability Factors

The best subset of predictors that emerged from the present statistical analyses, for both truth discernment and overall belief, points to a “contaminated mindware” (conspiracy mentality, anti-science attitude, and paranormal beliefs) factor [29] (i.e., the MIS and the FNSS conspiracy belief scores), to the right-wing preference for traditional social norms (RWAS; see also [30]), and to an over-claiming dimension (FNSS self-belief; [7]). These vulnerability factors replicate previous findings.

### 4.3. Protective Factors

The most interesting result concerning the protective factors is that higher scores on two subscales of RWA were associated with lower levels of overall belief for the COVID-19 news. However, this result cannot be interpreted as indicating that RWA acts as a protective factor; instead, it should be interpreted as the effect of partisan bias. The effect of age on truth discrimination also deserves a comment. Whereas previous reports indicate that older adults are especially susceptible to FN [31], we found the opposite result. However, our results are better understood if we consider the distribution of age in the present total sample: 26.8% young (0–20), 48.7% early adulthood (20–40), 23.1% middle adulthood (40–65), and only 1.3% old age (65–78). In the light of these data, therefore, the variable “age” should not be interpreted as indicating “cognitive decline”, but rather as suggesting the presence of suboptimal online information literacy among younger participants [32].

### 4.4. Implications of Present Results for Cognitive Interventions

Our results provide very strong evidence against the idea that an attentional focus on accuracy may be an effective intervention to counter misinformation online [7]. It is indisputable that during the Italian national lockdown, the issue of veracity of the COVID-19 news was a central aspect of the personal every-day experience of the large majority of the Italian population. In these conditions, the idea to ask individuals to “pay more attention to the accuracy of COVID-19 news” is completely futile: Every sentient Italian adult was already doing exactly that, to the best of her/his ability. Nevertheless, we found the same distortions on truth discernment as for low-personal-relevance topics.

The dependence of FN susceptibility on a cognitive deficit can be also questioned in terms of a Bayesian thinking framework. Paradoxically, in fact, the susceptibility to COVID-19 FN could be construed as a rational solution to the problem of finding valid information, conditional to the specific situation of some individuals.

The Italian national lockdown was characterized by an extreme information ambiguity, with contradictory and highly polarized COVID-19 news on all Italian major media outlets. Italians were provided with a paucity of believable information about which behavioral recommendations were appropriate for personal protection against the COVID-19 virus. According to Bayesian thinking, when the data (likelihood) are weak, the posterior beliefs are dominated by the prior. In Italy, the individuals who are particularly susceptible to misinformation often share a belief system (prior knowledge) characterized by exactly those elements on which the FN are mainly built (i.e., conspiratorial beliefs, institutional mistrust, epistemic beliefs that knowledge is fixed and unchangeable, magical thinking, and so on [33,34]). Therefore, when the available data are weak, such individuals perform a rational choice by falling back on their prior knowledge. It is unfortunate that, for these individuals, previous knowledge was completely misleading. But the way in which they combined the available information with their previous knowledge seems to be the best that anybody, *in a similar situation*, could do. The point is that FN vulnerability during the Italian lockdown cannot be ascribed to a cognitive information-processing deficit.

It is possible to arrive at the same conclusion, with reference to individuals prone to online misinformation, when considering the role of emotional factors. During the lockdown, the prevalent state in the Italian population was a feeling of threat [34]. Fear is a response to threat. In a situation of crisis, uncertainty, and fear, a defensive reaction leads individuals to strengthen the credibility of information consistent with their prior beliefs (confirmatory bias). For example, Van Bavel et al. [5] found that during the COVID-19 pandemic individuals showed the tendency to discredit information contradicting their prior beliefs. Again, it is not necessary to postulate a cognitive deficit.

A third speculation points to the role of social factors. In a situation of social conflict and danger, it is natural to believe the news shared by “friends” whom one can trust, rather than the news provided by the state-run media or by scientists who cannot agree with each other and, on a daily basis, propose opposite opinions on the media. If the friends of at-risk individuals share a belief system compatible with the FN (i.e., a belief system based on conspiratorial beliefs, institutional mistrust, bizarre epistemic beliefs, magical thinking, and so on), then at-risk individuals will fall prey to misinformation. Once again, there is no need to postulate a cognitive deficit.

These three speculations, rather than describing FN vulnerability in terms of a cognitive deficit, point to the importance of prior beliefs in response to a situation of crisis, threat, and uncertainty. Our data, together with the speculations outlined above, suggest that “cognitive interventions” may be ineffective in countering the effect of FN. Instead, the previous considerations point to the importance of interventions that may contribute to the development of a more mature (prior) belief system—which is tantamount to saying that is necessary to increase the level of instruction in the most vulnerable sectors of society.

### 4.5. Limitations

There are a number of potential limitations of this study that require consideration. The present study relies on a convenience sample which is not nationally representative and our comparisons of the personal relevance of news topics should be interpreted accordingly. In this respect, however, we repeat the consideration provided by Pennycook and Rand [8]: In a study on news truth discrimination and overall belief, obtaining a nationally representative sample may be less important than sampling from frequent internet and social media users, who are most likely to be exposed to FN. According to this point of view, therefore, the present sampling method has several advantages relative to a nationally representative sample.

Examining not only news veracity discrimination and overall belief, but also the behavioral ramifications of encountering FN is crucial. For instance, Greene and Murphy [33] discovered that a single exposure to FN regarding COVID-19 had a discernible, though minor, impact on subsequent behavioral intentions. Furthermore, they observed no influence on health behavior intentions when participants were given a generic warning about the perils of online misinformation concerning COVID-19. Extending beyond mere behavioral intentions, it is imperative to evaluate the downstream effects of misinformation on tangible behavior.

## 5. Conclusions

The prevailing literature suggests that personal relevance may diminish susceptibility to fake news by engaging more cognitive resources. Contrarily, our study contends this assertion, showing that both protective and vulnerability factors influence news truth discrimination equivalently across topics of high and low personal relevance. Indeed, these factors impact the discrimination between true and false news similarly regardless of the personal relevance attributed to the news topics. This insight calls for a re-evaluation of current intervention strategies, suggesting a need for a deeper understanding of the factors underlying news truth discrimination to effectively address the challenges of misinformation.

## Data Availability

We have made the data and R scripts necessary to run the present statistical analysis available online as a snakemake workflow (https://github.com/ccaudek/fakenews-anonymous; accessed on 2 September 2023.

## Supplementary Materials

The following supporting information can be downloaded at: https://www.mdpi.com/article/10.3390/bs13110896/s1. File S1: Guidelines for FN corpora construction; References [1,2,6,7,8,10,18,19,20,22,23,35,36,37,38,39,40,41,42,43,44,45,46,47,48,49,50,51,52,53,54,55,56,57,58,59].

## References

[1] Shu K., Sliva A., Wang S., Tang J., Liu H. (2017). Fake news detection on social media: A data mining perspective. Explor. Newsl..

[2] Lazer D.M., Baum M.A., Benkler Y., Berinsky A.J., Greenhill K.M., Menczer F., Zittrain J.L. (2018). The science of fake news. Science.

[3] Walter N., Cohen J., Holbert R.L., Morag Y. (2020). Fact-checking: A meta-analysis of what works and for whom. Polit. Commun..

[4] Pennycook G., Rand D.G. (2020). The psychology of fake news. Trends Cogn. Sci..

[5] Van Bavel J.J., Pereira A. (2018). The partisan brain: An identity-based model of political belief. Trends Cogn. Sci..

[6] Bronstein M.V., Pennycook G., Bear A., Rand D.G., Cannon T.D. (2019). Belief in fake news is associated with delusionality, dogmatism, religious fundamentalism, and reduced analytic thinking. J. Appl. Res. Mem. Cogn..

[7] Pennycook G., Rand D.G. (2020). Who falls for fake news? The roles of bullshit receptivity, overclaiming, familiarity, and analytic thinking. J. Pers..

[8] Pennycook G., Rand D.G. (2019). Lazy, not biased: Susceptibility to partisan fake news is better explained by lack of reasoning than by motivated reasoning. Cognition.

[9] Linden S van der Panagopoulos C., Roozenbeek J. (2020). You are fake news: Political bias in perceptions of fake news. Media Cult. Soc..

[10] Sindermann C., Cooper A., Montag C. (2020). A short review on susceptibility to falling for fake political news. Curr. Opin. Psychol..

[11] Petty R.E., Cacioppo J.T., Schumann D. (1983). Central and peripheral routes to advertising effectiveness: The moderating role of involvement. J. Consum. Res..

[12] Petty R.E., Cacioppo J.T., Goldman R. (1981). Personal involvement as a determinant of argument-based persuasion. J. Pers. Soc. Psychol..

[13] Jia L., Shan J., Xu G., Jin H. (2020). Influence of individual differences in working memory on the continued influence effect of misinformation. Cogn. Psychol..

[14] Stone A.R., Marsh E.J. (2023). Belief in COVID-19 misinformation: Hopeful claims are rated as truer. Appl. Cogn. Psychol..

[15] Rossi A.A., Panzeri A., Taccini F., Parola A., Mannarini S. (2022). The rising of the shield hero. Development of the Post Traumatic Symptom Questionnaire (PTSQ) and assessment of the protective effect of self-esteem from trauma-related anxiety and depression. J. Child Adolesc. Trauma.

[16] Sharot T., Sunstein C.R. (2020). How people decide what they want to know. Nat. Hum. Behav..

[17] Shahsavari S., Holur P., Wang T., Tangherlini T.R., Roychowdhury V. (2020). Conspiracy in the time of corona: Automatic detection of emerging COVID-19 conspiracy theories in social media and the news. J. Comput. Soc. Sci..

[18] Rubin V.L., Conroy N., Chen Y., Cornwell S. Fake news or truth? Using satirical cues to detect potentially misleading news. Proceedings of the Second Workshop on Computational Approaches to Deception Detection.

[19] Giuntoli L., Capuozzo P., Ceccarini F., Colpizzi I., Caudek C. (2020). Development and validation of the Fake News Supsceptibility Scale.

[20] Rattazzi A.M.M., Bobbio A., Canova L. (2007). A short version of the Right-Wing Authoritarianism (RWA) Scale. Pers. Individ. Differ..

[21] Sinclair A.H., Stanley M.L., Seli P. (2020). Closed-minded cognition: Right-wing authoritarianism is negatively related to belief updating following prediction error. Psychon. Bull. Rev..

[22] Eckblad M., Chapman L.J. (1983). Magical ideation as an indicator of schizotypy. J. Consult. Clin. Psychol..

[23] Martin J.G., Westie F.R. (1959). The tolerant personality. Am. Sociol. Rev..

[24] Bürkner P.C. (2017). brms: An R package for Bayesian multilevel models using Stan. J. Stat. Softw..

[25] Dasgupta S., Moore M.R., Dimitrov D.T., Hughes J.P. (2021). Bayesian validation framework for dynamic epidemic models. Epidemics.

[26] Pennycook G., Cheyne J.A., Barr N., Koehler D.J., Fugelsang J.A. (2015). On the reception and detection of pseudo-profound bullshit. Judgm. Decis. Mak..

[27] Spohr D. (2017). Fake news and ideological polarization: Filter bubbles and selective exposure on social media. Bus. Inf. Rev..

[28] Mourão R.R., Robertson C.T. (2019). Fake news as discursive integration: An analysis of sites that publish false, misleading, hyperpartisan and sensational information. J. Stud..

[29] Rizeq J., Flora D.B., Toplak M.E. (2021). An examination of the underlying dimensional structure of three domains of contaminated mindware: Paranormal beliefs, conspiracy beliefs, and anti-science attitudes. Think. Reason..

[30] Clarke E.J., Klas A., Dyos E. (2021). The role of ideological attitudes in responses to COVID-19 threat and government restrictions in australia. Pers. Individ. Differ..

[31] Sica C., Caudek C., Cerea S., Colpizzi I., Caruso M., Giulini P., Bottesi G. (2021). Health anxiety predicts the perceived dangerousness of COVID-19 over and above intrusive illness-related thoughts, contamination symptoms, and state and trait negative affect. Int. J. Environ. Res. Public Health.

[32] Sica C., Perkins E.R., Latzman R.D., Caudek C., Colpizzi I., Bottesi G., Patrick C.J. (2021). Psychopathy and COVID-19: Triarchic model traits as predictors of disease-risk perceptions and emotional well-being during a global pandemic. Pers. Individ. Differ..

[33] Greene C.M., Murphy G. (2021). Quantifying the effects of fake news on behavior: Evidence from a study of COVID-19 misinformation. J. Exp. Psychol..

[34] Rossi A.A., Marconi M., Taccini F., Verusio C., Mannarini S. (2021). From fear to hopelessness: The buffering effect of patient-centered communication in a sample of oncological patients during COVID-19. Behav. Sci..

[35] Vlachos A., Riedel S. Fact checking: Task definition and dataset construction. Proceedings of the ACL 2014 Workshop on Language Technologies and Computational Social Science.

[36] Klayman J. (1995). Varieties of confirmation bias. Psychol. Learn. Motiv..

[37] Altemeyer B. (1998). The other “authoritarian personality”. Adv. Exp. Soc. Psychol..

[38] Garzitto M., Picardi A., Fornasari L., Gigantesco A., Sala M., Fagnani C., Stazi M.A., Ciappolino V., Fabbro F., Altamura A.C. (2016). Normative data of the Magical Ideation Scale from childhood to adulthood in an Italian cohort. Compr. Psychiatry.

[39] Chapman L.J., Chapman J.P. (1987). The search for symptoms predictive of schizophrenia. Schizophr. Bull..

[40] Carlucci L., Tommasi M., Saggino A. (2013). Factor structure of the Italian version of the religious fundamentalism scale. Psychol. Rep..

[41] R Core Team (2021). R: A Language and Environment for Statistical Computing.

[42] Wickham H., Averick M., Bryan J., Chang W., McGowan L.D., François R., Grolemund G., Hayes A., Henry L., Hester J. (2019). Welcome to the tidyverse. J. Open Source Softw..

[43] Bürkner P.-C. (2018). Advanced Bayesian multilevel modeling with the R package brms. R J..

[44] Green D.M., Swets J.A. (1966). Signal Detection Theory and Psychophysics.

[45] Metzger M.J., Hartsell E.H., Flanagin A.J. (2020). Cognitive dissonance or credibility? A comparison of two theoretical explanations for selective exposure to partisan news. Commun. Res..

[46] Ross R.M., Rand D.G., Pennycook G. (2021). Beyond “fake news”: Analytic thinking and the detection of false and hyperpartisan news headlines. Judgm. Decis. Mak..

[47] Zrnec A., Poženel M., Lavbič D. (2022). Users’ ability to perceive misinformation: An information quality assessment approach. Inf. Process. Manag..

[48] Gelman A., Goodrich B., Gabry J., Vehtari A. (2019). R-squared for bayesian regression models. Am. Stat..

[49] Stagnaro M., Pennycook G., Rand D.G. (2018). Performance on the cognitive reflection test is stable across time. Judgm. Decis. Mak..

[50] Engelhardt A.M., Feldman S., Hetherington M.J. (2021). Advancing the measurement of authoritarianism. Political Behav..

[51] Meyer T.D., Hautzinger M. (1999). Two-year stability of psychosis proneness scales and their relations to personality disorder traits. J. Personal. Assess..

[52] Lai K., Xiong X., Jiang X., Sun M., He L. (2020). Who falls for rumor? Influence of personality traits on false rumor belief. Personal. Individ. Differ..

[53] Brashier N.M., Schacter D.L. (2020). Aging in an era of fake news. Curr. Dir. Psychol. Sci..

[54] Pennycook G., Cheyne J.A., Koehler D.J., Fugelsang J.A. (2020). On the belief that beliefs should change according to evidence: Implications for conspiratorial, moral, paranormal, political, religious, and science beliefs. Judgm. Decis. Mak..

[55] Alper S., Bayrak F., Yilmaz O. (2020). Psychological correlates of COVID-19 conspiracy beliefs and preventive measures: Evidence from turkey. Curr. Psychol..

[56] Piironen J., Paasiniemi M., Vehtari A. (2020). Projective inference in high-dimensional problems: Prediction and feature selection. Electron. J. Stat..

[57] Vehtari A., Gelman A., Gabry J. (2017). Practical bayesian model evaluation using leave-one-out cross-validation and WAIC. Stat. Comput..

[58] Gelman A., Hill J., Vehtari A. (2020). Regression and Other Stories.

[59] Skitka L.J., Mullen E., Griffin T., Hutchinson S., Chamberlin B. (2002). Dispositions, scripts, or motivated correction? Understanding ideological differences in explanations for social problems. J. Personal. Soc. Psychol..

