# Supplementary material for: Breaking (Fake) News: No Personal Relevance Effect on Misinformation Vulnerability
Source: Behav Sci (Basel). 2023 Oct 30;13(11):896. doi: 10.3390/bs13110896 (PMC10669344; doi:10.3390/bs13110896)
Supplement: Supplementary file 1 [file behavsci-13-00896-s001.zip › behavsci-2657804-supplementary.pdf]

Supplementary Materials: Appendix

Breaking (Fake) News: No Personal Relevance Effect on Misinformation Vulnerability

Francesco Ceccarini [1], Pasquale Capuozzo [2], Ilaria Colpizzi [3] and Corrado Caudek [3,*]

[1] New York University Abu Dhabi, United Arab Emirates; fc2284@nyu.edu

[2] IRCCS Istituto Giannina Gaslini, Genoa, Italy; pasqualecapuozzo@gaslini.org

[3] University of Florence, Italy; ilaria.colpizzi@unifi.it

* Correspondence: corrado.caudek@unifi.it

Guidelines for FN corpora construction

According to [1], a FN dataset should: (1) include both fake and real news items, (2) contain text-only news items, (3) have a verifiable ground-truth, (4) be homogeneous in length and (5) writing style, (6) contain news from a predefined time frame, (7) be delivered in the same manner and for the same purpose (*e.g.*, humor, breaking news) for fake and true cases, (8) be made publicly available, and (9) should take language and cultural differences into account. Among these criteria, ground-truth verification (fact-checking) requires the assessment of the truthfulness of claims made by public figures such as politicians and pundits [35], and represents the core process of news truthfulness evaluation. When collecting data for research purposes, a common practice for such criterion is to rely on veracity judgements provided by qualified fact-checking platforms.

This procedure resulted in two datasets composed of 30 items each, satisfying all the criteria provided by [18].

Headline news corpora

We generated two sets of headlines, each containing 15 FN and 15 TN. Each news was represented only with its original textual headline. All headlines were comparable in length and writing style. Ground-truth checking relied on reliable sources and fact-checking platforms.

- FN about COVID-19 were collected from 7 February to 4 April 2020. TN about COVID-19 were collected from 6 March to 11 April 2020.
- FN and TN about politics were collected from 7 March to 31 May 2021.

All these news are publicly available online. Headline news were in Italian, because news credibility was judged by two samples of Italian speaking participants.

TN headlines were recovered from institutional websites such as the National Health Institute and the Ministry of Health, from national media websites reporting citing the source, or from scientific papers. To assess the eligibility of FN to be part of our corpora, we consulted the following fact-checking platforms: Bufale, Butac, Pagella Politica, Factcheck, and the fact-checking section of Open Online.

Fake News (FN) and True News (TN) Dataset: Covid-related news, March 2020 – May 2020

Fake News (FN)

FN1.

Martin Pall: those who stay away from 5G will escape the Coronavirus (Italian version: Martin Pall: sfuggirà al Coronavirus chi resta lontano dal 5G) is a FN, as discussed by J. Pili in her article on the website www.openonline.com. There is no scientific evidence of a possible association between 5G and COVID-19.

FN2.

Coronavirus, beer scares the USA: 38% would not buy Corona (Ita version: Coronavirus, la birra spaventa gli Usa: 38% non comprerebbe Corona) is a false news, as proved by an article published on the fact-checking site www.pagellapolitica.it, there is no evidence of a decrease in Corona beer consumption during the pandemic.

FN3.

The news *Boldrini: self-certification must be change: the female adjective is missing on the Coronavirus forms (Ita version: Boldrini: vuole cambiare l'autocertificazione: manca il femminile sui moduli per il Coronavirus)* was denied, as it is a distortion of a statement made by the Italian MP Laura Boldrini, as proven by David Puente in his article published on the website www.openonline.com

FN4.

The news *Is there the Coronavirus? 2000 dogs were abandoned (Ita version: C'è il Coronavirus? Abbandonati 2mila cani)* proved to be fake, as we learn from an article published by the pseudonym "maicolengel butac" on the fact-checking site www.butac.it, there is no evidence of abandoned dogs due to COVID-19.

FN5.

The news *Ronaldo fights the Coronavirus: his hotels will become hospitals (Ita version: Ronaldo combatte il coronavirus: i suoi hotel diventeranno ospedali)* turned out to be false, since, as reported by the factchecking site www.bufale.net, there is no evidence that the hotels owned by the football player Cristiano Ronaldo were being used as hospitals.

FN6.

Chinese scientists: Coronavirus came out of a laboratory near the Wuhan market (Ita version: Scienziati cinesi: Il Coronavirus è uscito da un laboratorio vicino al mercato di Wuhan) is a FN, as reported by J. McDonald in her article published on the website www.factcheck.org, there is no scientific evidence that the Coronavirus has been bioengineered.

FN7.

Coronavirus, clean and change clothes to prevent contagion: here is how often and how (Ita version: Coronavirus, lavare e cambiare abiti e vestiti per prevenire il contagio: ecco ogni quanto e come) is a false news, without any scientific basis as reported on the website of the Istituto Superiore di Sanità (www.iss.it/) “If the distance of at least one meter from other people is observed, it is unlikely that our clothes, or ourselves, can be contaminated with viruses in significant quantities".

FN8.

The hot lemon can remove the virus from our body (Ita version: Il limone caldo può uccidere la proliferazione di questo virus nel nostro organismo) is a false information, as reported by David Puente in his article published on the website www.openonline.com, there is no scientific evidence that the hot lemon may defeat the Coronavirus.

FN9.

The news *Italy arrests a doctor for the intentional killing of over 3,000 Coronavirus patients (Ita version: L'Italia arresta un medico per l'uccisione intenzionale di oltre 3.000 pazienti con Coronavirus)* is false, as can be seen from the article by David Puente published on www.openonline.com, there was no such case in Italy.

FN10.

The news *The shock "contract" to the elderly: "Signed to refuse treatment" (Ita version: Il "contratto" choc agli anziani: "Firmate per rifiutare le cure")*, which refers to Holland, is false, and it can be learned from an article on www.butac.it, there is no evidence of such agreement in the Netherlands.

FN11.

Government interrupts private property until July 31: any movable and immovable property can be confiscated (Ita version: Governo sospende proprietà privata fino al 31 Luglio: ogni bene mobile e immobile potrà essere requisito) is a FN, as revealed in the article of www.bufale.net, which highlights that this information is an intentional distortion of the 7th paragraph of the *Cura Italia* act.

FN12.

The news *Coronavirus: Government already knew everything since January 31 but kept silent, devastating Italy. Here is the evidence (Ita version: Coronavirus: il governo sapeva tutto già il 31 gennaio ma ha taciuto devastando l'Italia. Ecco le prove)* is a false information, as reported in the article by David Puente published on www.openonline.com, the Government has declared a state of emergency since January 31 and this information was accessible to public opinion and was not kept hidden.

FN13.

Coronavirus, doctor confesses: the Government prevents us from swabbing migrants on ONGs (Ita version: Coronavirus, medico confessa: il Governo ci impedisce di fare i tamponi ai migranti sulle ONG) is a FN, as reported by David Puente in his article published by the website www.openonline.com, which informs that these statements are false, no doctors have stated such claims.

FN14.

The news *In Italy, there were 2000 artificial respirators, but the Government waited a month to look for them (Ita version: C'erano duemila respiratori in Italia, ma il governo ha aspettato un mese per cercarli)* is a false information, as reported in the article released by www.butac.it, it was about two-thousand ventilators that had to be produced rather than being ready for use.

FN15.

Ibuprofen can aggravate COVID-19 infection (Ita version: Ibuprofene può aggravare l'infezione da COVID-19) is a FN, as reported on the website www.factcheck.org, "there is currently no evidence that ibuprofen increases the risk of serious complications or of acquiring the virus that causes COVID-19" and the World Health Organization has clearly stated that there are no contraindications to taking medications containing Ibuprofen (www.who.int/).

True News (TN)

TN1.

Coronavirus: tap water is not dangerous. The Istituto Superiore di Sanità (ISS) explains why the mineral water is not necessary (Ita version: Coronavirus: l'acqua del rubinetto non è pericolosa. L'Istituto Superiore di Sanità (ISS) spiega perché non serve la minerale) is a TN, as reported by the ISS:" The current purification practices are effective

in killing the virus, given the retention times and dilution phenomena that characterize the treatments, combined with environmental conditions that compromise the viability of the viruses (temperature, sunlight, high pH levels) ".

TN2.

The news *WHO: Pets do not transmit the virus but can be infected (Ita version: OMS: Animali domestici non trasmettono ma possono essere contagiati)* is well founded. Maria Van Kerkhove, head of the WHO emerging diseases and zoonosis units, stated that "at the moment there is no evidence that pets could transmit the Coronavirus to human but experts found that pets might be infected by their owners (www.salute.gov.it).

TN3.

The news *Coronavirus, Arcuri:"Only thanks to vaccine the emergency will be overcome" (Ita version: Coronavirus, Arcuri: "Emergenza sarà definitivamente superata solo con vaccino")* is true. Domenico Arcuri, at the time extraordinary commissioner for the strengthening of hospital infrastructures necessary to cope with the COVID-19 emergency, actually released such statement to the media, as reported in an article by Francesco Giovannetti on the website of the newspaper "La Stampa" (www.lastampa.it).

TN4.

The news *Coronavirus, the army brings 60 coffins out of Bergamo (Ita version: Coronavirus, l'esercito porta 60 bare da Bergamo fuori regione)* is dramatically true, as stated by the Mayor of the city of Bergamo, Giorgio Gori. The article on the website www.tgcom24.mediaset.it showed the real images of the fact attached to the Mayor's words.

TN5.

Coronavirus, the appeal of veterinarians:"Do not use bleach to sanitize the dogs' paws" (Ita version: Coronavirus, l'appello dei veterinari: "Non usate la candeggina per disinfettare le zampe dei cani) is a TN, as it was stated by Luca Robutti, president of the Order of Veterinary Doctors of the Savona province, in his interview by Arianna Codato on the website of the newspaper "La Stampa" (www.lastampa.it): bleach could cause irreversible damage to the pets' paws.

TN6.

The news *Coronavirus, there is no evidence that food is a source or vehicle of transmission of the virus (Ita version: Coronavirus, non ci sono prove che il cibo sia fonte o veicolo di trasmissione)* is a true information, as reported on the website of the "European Food Safety Authority" (www.efsa.europa.eu) issued by Marta Hugas, the scientific director EFSA: "Previous experiences with other coronavirus outbreaks, such as SARS-CoV and MERS-CoV, showed that no transmission through food consumption had occurred. There is currently no evidence that COVID-19 can be different in any way."

TN7.

The news *COVID-19, Gimbe:" Social distancing measures are effective in holding the spread (Ita version: Misure di distanziamento sociale efficaci per contrastare la diffusione)* is real, as it is clearly stated by Nino Cartabellotta, president of the "Gimbe Foundation", whose purpose is to promote and implement training and research activities in the health sector: "The infections spread mainly through close contact between people, therefore, in the absence of a COVID-19 vaccine, social distancing measures are the the only weapon to fight this pandemic" (article published on the website www.rifday.it).

TN8.

The news *All ages contribute to the spread of the infection (Ita version: Tutte le fasce di età contribuiscono alla propagazione dell'infezione)* is true, as it emerged from an investigation of the ISS in which they showed that no age is immune from the virus (www.iss.it).

TN9.

COVID-19, the smokers are more at risk of getting the infection (Ita version: COVID-19, fumatori più a rischio di contrarre l'infezione) is a TN, as reported on an article published on the website of the Ministry of Health (www.salute.gov.it). Here are listed the evidences in support of this statement.

TN10.

The news *Incubation and Coronavirus: symptoms appear on average 5 days after infection (Ita version: Incubazione e coronavirus: i sintomi compaiono in media dopo 5 giorni dall'infezione)* is well founded, as evidenced by Mara Magistroni in her article published on www.wired.it, which summarizes the principle results of a recent study made by a team from the Johns Hopkins Bloomberg School of Public Health and published in the Annals of Internal Medicine Journal.

TN11.

The news *The World Health Organization declares that the Coronavirus is a pandemic (Ita version: L'organizzazione mondiale della sanità dichiara il Coronavirus pandemia)*, as we all remember, is a a true information. The statement by the WHO General Director Tedros Adhanom Ghebreyesus was reported on the media of the whole world, in Italy is available on the website of the Ministry of Health (www.salute.gov.it).

TN12.

The Coronavirus symptoms are not just coughs and respiratory crises. Here are the rarest symptoms, from diarrhea to vomiting (Ita version: Il coronavirus non provoca solo tosse e crisi respiratorie. Ecco i sintomi più rari, dalla diarrea al vomito) is a TN, the website www.it.businessinsider.com reported the words of several doctors, who pointed out that the symptoms of their COVID-19 patients included other symptoms besides coughing and respiratory crisis.

TN13.

The news *C vitamin is not a cure for the Coronavirus (Ita version: La vitamina C non cura il Coronavirus)* is true, as reported on the website www.humanitas.it, there is no scientific evidence that C vitamin (ascorbic acid) can cure the Coronavirus or prevent its contagion.

TN14.

The news *Mosquito bites do not transmit the coronavirus (Ita version: Le punture delle zanzare non trasmettono il coronavirus)* is true, as reported in an article by Andrea Centini published on www.fanpage.it, in which Fabrizio Pregliasco, virologist at the Department of Biomedical Sciences for the Health of the University of Milan, exposed various scientifically based arguments in support of this statement.

TN15.

WHO confirms: Coronavirus is not transmitted by air, but only through droplets of saliva (Ita version: L'OMS conferma: il coronavirus non si trasmette per via aerea, ma solo tramite le goccioline di saliva) is a TN, discussed on the official website of the WHO (www.who.int/) within the scientific summary "Ways of virus transmission responsible for the COVID-19 spread: Implications for the IPC precautionary recommendations", where it is specified that "Air transmission is different from droplet transmission, because the droplet transmission

consists of the presence of microbes inside the nuclei of droplets, which can remain in the air for a long time and be transmitted to others for distances greater than 1 m".

Fake News (FN) and True News (TN) Dataset: Political-related news, March 2021 – May 2021

Fake News (FN)

FN1.

Boldrini asks Draghi for a "dignity salary" for migrants (Ita version: Boldrini chiede a Draghi un "reddito di dignità" per i migranti) is a FN, as reported on the website www.facta.news. The news is a distortion of a statement made by the Italian MP Laura Boldrini in an interview with the newspaper "La Repubblica" on December 22, 2015.

FN2.

Lamorgese closes road toll booths to stop the restaurateurs' march on Rome: "We will stop them!" (Ita version: Lamorgese chiude caselli stradali per fermare la marcia su Roma dei ristoratori: "Li fermeremo!") is a false news, reported on the website www.voxnews.info. The Interior Minister Luciana Lamorgese never closed the toll booths to stop demonstrations in Rome.

FN3.

The news *Lorenzo Fioramonti, the latest gimmick: one million a year for LGBT courses in schools (Ita version: Lorenzo Fioramonti, l'ultima trovata: un milione l'anno per i corsi Lgbt nelle scuole)* is a false information, as it is a distortion of a statement made by the Italian former Education Minister Lorenzo Fioramonti, who proposed funding for gender education to promote equality, not to for the LGBT world (www.butac.it).

FN4.

The news *The PD party wants "Bella Ciao" at school "Sing it with the Hymn of Mameli" (Ita version: Il PD vuole "Bella Ciao" a scuola "Cantatela con l'Inno di Mameli")* was denied, as reported on the website www.bufale.net, which proved that the information was manipulated. The PD party never proposed to sing the partisan song in schools.

FN5.

The abolition of the car tax and the Rai license fee is real (Ita version: Arriva l'abolizione del bollo auto. Cancellato anche canone Rai) is a FN, in Italy the two taxes, the Rai license fee and the car tax, have never been removed (www.bufale.net).

FN6.

Mini mandatory conscription, the OK from the Chamber: 6 months in barracks for young people between 18 and 22 years (Ita version: Mini leva obbligatoria, arriva l'ok dalla Camera: 6 mesi di caserma per giovani tra i 18 e 22 anni) is a FN, as explained on the website (www.bufale.net). The Parliament never approved such proposal.

FN7.

Fired for a selfie with Salvini but the judge accept the appeal (Ita version: Licenziato per un selfie con Salvini ma il giudice gli dà ragione) is a distorted information, as reported on the website www.bufale.net, which explained that the worker in question was fired for reasons not related to politics.

FN8.

The news *Refugee confesses: "I fled to Italy because I killed so many people" (Ita version: Profugo confessa: "Fuggito in Italia perché ho ucciso tante persone")* is false, as reported on the website www.bufale.net.

FN9.

Huge fines to all parents of bullying children (Ita version: Multe salate a tutti i genitori di ragazzi che compiono atti di bullismo) is a false information, as reported on the website www.bufale.net. The Italian law does not provide for any fine for bullying at school.

FN10.

The news *Christine Lagarde: "The elderly live too much and are dangerous for the world economy" (Ita version: Christine Lagarde: "Gli anziani vivono troppo e sono pericolosi per l'economia mondiale")* is false. No official source has reported such statement by the President of the European Central Bank, Christine Lagarde, as discussed on the website www.open.online.

FN11.

The news *Grillo shock: "if we win the elections, for those who voted PD there will be prison" (Ita version: Grillo shock: "se andiamo al governo per chi avrà votato PD ci sarà il carcere"* is a false information reported on the website www.ilquotidaino.wordpress.com. In fact, no official source has reported such statement by Beppe Grillo and the threatened measure has never been applied in Italy.

FN12.

The Government finances the foreign mafias with the citizenship salary: Explosion of subsidies to the mafia (Ita version: Il Governo finanzia le mafie straniere con il reddito di cittadinanza: Boom di sussidi ai mafiosi) is a FN published on the disinformation website www.voxnews.info. There is no evidence or accusations to support such statement.

FN13.

Macron: "Italy deserves all immigrants because it was a fascist country" (Ita version: Macron: "L'Italia merita tutti gli immigrati perché era un paese di fascista") is a FN, as reported on the website www.bufale.net. The french former Prime Minister Emmanuel Macron never claimed such statement, no official source has reported it, he had said instead "Italy deserves better leaders".

FN14.

The news *African kills and roasts a cat: PD Mayor hides the fact with the taxpayers money to avoid retaliations (Ita version: Africano ammazza gatto e lo arrostisce: Sindaco PD lo nasconde a spese dei contribuenti per evitare ritorsioni)* is a false information published on the website www.voxnews.info. As discussed by the www.bufale.net website, no politicians tried to cover such fact with or without the taxpayers money.

FN15.

Africans pass through dozens of European countries to be maintained in Italy (Ita version: Africani attraversano decine di paesi europei per farsi mantenere in Italia) is a FN published on the disinformation website www.voxnews.info. In fact, no official source has reported evidence or accusations to support such statement.

True News (TN)

TN1.

Italian Parliamentarians have the highest salaries in the world: An EU study says so: "140.000 euros to the Italians, 90.000 to the Germans" (Ita version: I parlamentari italiani hanno gli stipendi più alti del mondo: ora lo dice anche uno studio dell'UE: "140mila euro, ai tedeschi 90mila") is a TN discussed in an web-article published by the newspaper "Il Fatto Quotidiano" (www.ilfattoquotidiano.it). The article showed results of the comparative

study made by Brussels: Italian deputies and senators earn on average 40.000 euros more than their German counterparts, 56.000 euros more than the French, 35.000 more than the Americans, double the English lords and 10 times more than the Hungarians.

TN2.

The news *Malika speaks, kicked out from home because she is a lesbian: " Don't be ashamed for who you are and who you love " (Ita version: Parla Malika, cacciata di casa perché lesbica: "Non vergognatevi per chi siete e per chi amate")* is real, as discussed by many journals and media in Italy, the story of Malika had a wide media coverage. It is possible to read the entire article on the website of the newspaper "La Repubblica" (www.repubblica.it/).

TN3.

"Confused the taser for the gun" so the police killed 20-year-old African-American Daunte Wright (Ita version: "Scambiato il taser con la pistola" così la polizia ha ucciso il 20enne afroamericano Daunte Wright) is a TN, as discussed in many official sources, such as the website www.fanpage.it and www.ilfattoquotidiano.it. On April 21, 2021, in Minneapolis, former police officer Kim Potter shot the 20-year-old African-American Daunte Wright.

TN4.

The news *Von Der Leyen without a chair at the meeting with Erdogan. Ankara's rudeness becomes a diplomatic case (Ita version: Von Der Leyen senza poltrona all'incontro con Erdogan. Lo sgarbo di Ankara diventa un caso diplomatico)* is real and very discussed. The news had a wide media coverage. As reported on the website www.ilsole24ore.com, on April 6, 2021, the EU leaders are visiting Ankara, in the presence of Erdogan. The Turkish president welcomed the President of the EU Council, Charles Michel, as per protocol rules, but denied the same hospitality to the president of the European Commission, Ursula von der Leyen.

TN5.

Trump supporters assault the Capitol Hill: 4 dead. Weapons in the chamber. The National Guard intervenes. Biden: "The president must stop this attack to democracy" (Ita version: Sostenitori di Trump assaltano il Campidoglio: 4 morti. Armi in aula. Interviene la Guardia Nazionale. Biden: "Il presidente fermi questo attacco alla democrazia") is a TN, as reported in a web-article of the newspaper "La Stampa" (www.lastampa.it). After the former US president Donald Trump lost the election against Biden, his supporters attacked the Capitol Hill in protest. The fact was unprecedented.

TN6.

The news *Prince Philip, Duke of Edinburgh died: he was 99 years old (Ita version: È morto il principe Filippo, duca di Edimburgo: aveva 99 anni)* is real and verifiable. On April 9, 2021, many newspapers and websites reported the news of the prince Philip death (www.corriere.it).

TN7.

USA, constituency confirms Biden's victory (Ita version: Usa, il collegio elettorale conferma la vittoria di Biden) is a TN. As reported on the website www.ansa.it, the constituency confirmed Joe Biden's victory with 306 votes, against Donald Trump's 232 (the quorum to be elected president is 270 electors). "The victory is clear," "the will of the people prevailed," Biden said.

TN8.

The news *Rome, tensions and clashes with the police at the "Io Apro" demonstration (Ita version: Roma, tensioni e scontri con la polizia alla manifestazione "Io Apro")* is true, as reported on the website www.tgcom24.mediaset.it, on April 12, 2021, many freelancers and owners demonstrated in Rome against

the mandatory closures of commercial establishments. The demonstration was pacific, however there were some clashes.

TN9.

Draghi defines Erdogan a dictator, Turkey summons the Italian ambassador: "Very serious words" (Ita version: Draghi definisce Erdogan un dittatore, Turchia convoca l'ambasciatore italiano: "Parole gravissime") is a TN, as discussed on the website www.fanpage.it. The Italian Prime Minister Mario Draghi said, during a press conference, that he absolutely disagrees with Erdogan's behavior towards the president of the European Commission, Ursula Von der Leyen and he defined Ergogan "a dictator". After such declaration, Turkey summoned the Italian ambassador to Ankara. Harsh response from Turkish Foreign Minister, Mevlut Cavusoglu: "We strongly condemn the uncontrolled claims of Italian Prime Minister Mario Draghi about our elected president".

TN10.

Poland, thousands of women demonstrating in the streets of Warsaw against the law that prohibits abortion (Ita version: Polonia, migliaia di donne in piazza a Varsavia contro la legge che vieta l'aborto) is a TN, as reported by many official source, such as www.corriere.it and www.lastampa.it, that discussed what happened in Poland this year. Thousands of people remonstrated against the sentence of the Constitutional Court which denied abortion even in case of the fetus malformations.

TN11.

The news *Liliana Segre presents in Senate to vote for Patrick Zaki Italian citizenship (Ita version: Liliana Segre in Senato per il voto sulla cittadinanza italiana a Patrick Zaki)* is real, as reported in a web-article of the newspaper "Il Fatto Quotidiano" (www.ilfattoquotidiano.it), the life-long senator Liliana Segre was presented in Senate

to vote for Patrick Zaki Italian citizenship, the Egyptian student arrested in Egypt for his political ideas and commitment to human rights.

TN12.

The news *Renzi to Prince Bin Salman: "Saudi Arabia is the center of a new Renaissance" (Ita version: Renzi al principe Bin Salman: "Arabia Saudita centro di un nuovo Rinascimento")* is true, as reported on the website www.tpi.it. With this statement, the Italian MP Matteo Renzi opened his speech to the 4th edition of the "Future Investment Initiative"(FII) conference, also known as the "Davos of the desert", dedicated this year to the theme of the "neo-Renaissance".

TN13.

Senator Formigoni will receive the annuity as a member of Parliament (Ita version: Il senatore Formigoni riprenderà a ricevere il vitalizio da parlamentare) is a TN, as reported in a web-article of the newspaper "Il Fatto Quotidiano" (www.ilfattoquotidiano.it). The fact was very discussed in Italy, because Formigoni was a former president definitively sentenced for corruption, however the Senate decided to restore his salary as a parliamentary.

TN14.

Di Battista: "I leave the M5s, I do not accept a Government with these parties" (Ita version: Di Battista: "Lascio il M5s, non accetto un governo con questi partiti") is a TN, after the decision of the M5s to approve the Draghi Government, the Italian MP Di Battista himself declared that he will leave the party, as reported in the website of the newspaper "La Repubblica" (www.repubblica.it). As everyone can see, his name is no longer present on the Rousseau platform of his party.

TN15.

The news *Letta: "Pd encrusted with machismo, we need shock treatment" (Ita version: Letta: "Pd incrostato di maschilismo, serve cura choc")* is real, as reported on the website www.tgcom24.mediaset.it, the Italian MP Enrico Letta stated that more female figures are necessary in politics.

Procedure

Participants were recruited online on a voluntary basis using an advertising-based strategy through posts to popular social media sites and targeted e-mail lists. Individuals were considered eligible to participate in the study if they (1) were 18 years or older, (2) spoke Italian as their primary language, and (3) did not have any psychiatric illness or any alcohol/substance use disorder. After reading and digitally signing the informed consent, the participants provided demographic information (including age, sex, education, marital status, occupational status, political ideology with respect to economic and social issues, and media consumption habits (weekly time spent reading newspapers, reading news online, sharing news on social media, and so on). Then they were asked to judge 30 headlines (15 TN, 15 FN). For each headline (either about COVID-19 related news or about political related news), participants responded to the question "Based on the data in your possession, how much are you willing to believe the following news?" on a 5-point forced-choice confidence scale ranging from "Extremely confident that the news is true" to "Extremely confident that the news is fake". The presentation order of the headline news was randomized for ground-truth. Each news headline was shown until the participant's response and then removed. The response scale was presented together with each headline news until the participant's response. No feedback was presented to indicate whether the news was true or fake. Finally, participants compiled four self-report questionnaires. The data collection concerning the COVID-19 news took place during the forced national Italian lockdown (from 28 April to 12 May 2020). The mandatory quarantine period lasted from 9 March to 3 June 2020. The data collection regarding the political news took place from 7 March to 31 May 2021. Participants did not receive any monetary incentive for taking part in the study. No limitation in time was allotted for completing the task; only a single submission per participant was allowed.

External measures

For all the participants, we measured the following psychometric scales.

- *Fake News Susceptibility Scale* (FNSS): is a 16-item self-report scale assessing individual propensity to believe in news articles that are actually false [19] and is composed of four sub-scales: Irrational beliefs (*e.g.*, "It is possible to communicate with the dead"), Conspiratorial beliefs (*e.g.*, "Few powerful individuals secretly rule the world"), Critical news consumption (*e.g.*, "I like to try to understand if what I read or hear is true"), and Self belief [or self-confirmation bias: the tendency to favor information that confirms or supports one's prior personal beliefs, while overlooking opposing views; [36]; *e.g.*, "The opinion of others hardly makes me change my mind on a subject"]. To the best of our knowledge, the FNSS is the only self-report questionnaire applicable to the Italian population and measuring vulnerability to FN. In the present study, the ordinal (Ω) coefficients were 0.80 (Irrational beliefs) 0.83 (Conspiratorial beliefs), 0.72 (Critical news consumption), and 0.70 (Self-beliefs); total reliability was Ω = 0.82.

- *Right-Wing Authoritarianism Scale* (RWAS): since the first formulation by [37], right-wing authoritarianism has been conceptualized in terms of two linked clusters: authoritarian submission and aggression (willingness to comply with authority and support for authority-sanctioned violence toward minorities), and conventionalism (acceptance of traditions and social norms). We employed the 14-items short version of the RWA [20]. In the present study, internal consistency was Ω total = 0.86 ($\Omega_{s,a}$ = 0.86; Ω_c = 0.77).

- *Magical Ideation Scale* (MIS): originally developed by [22], translated and validated in Italian by [38], the MIS is a 30-item true-false scale measuring Magical ideation (*e.g.*, "I have wondered whether the spirits of the dead can influence the living"). The MIS highlights a wide range of attenuated

forms of thought-transmission experiences and aberrant beliefs such as astrology, thought withdrawal, spirit influences, conspiracy theories, UFOs, reincarnation, or good luck charms [39]. In the present sample, Ω = 0.89.

- *Religious Fundamentalism Scale* (RFS): devised by [23], the RFS is a 9-item self-report questionnaire measuring the degree of adherence to religious fundamentalism (*e.g.*, "If more of the people in this country would turn to Christ we would have a lot less crime and corruption"). The RFS has been validated for applications to the Italian population [40] and is composed of two negatively correlated factors identifying orthodox attitudes (Belief sub-scale) and rejection of religious dogma (Skepticism sub-scale). The internal consistency of the Italian version is Ω = 0.84 (Belief sub-scale) and Ω = 0.79 (Skepticism sub-scale). In the present study, the Ω coefficients were 0.87 (Belief) and 0.82 (Skepticism).

Demographic information

The COVID-19 news sample comprised 651 individuals with a mean age of 31.3 years (SD = 13.6); 72.5% were female. The average level of education was 14.0 years (*SD* = 2.40). Marital status was: 24.1% married, 9.7% cohabitation, 63.3% not married/no cohabitation, 2.6% divorced, 0.5% widows/widowers. Occupational status was: 7.0% unemployed seeking first or new job, 56.1% not in labor force, 37.0% full-time or part-time employee. A total of 460 participants were recruited in the political-news administration. They were on average 31.5 years old (SD = 14.4) and the 66.1% were female. Their average level of education was 14.20 years (*SD* = 2.35). Marital status: 21.3% married, 6.7% cohabitation, 67.4% not married/no cohabitation, 3.5% divorced, 1.1% widows/widowers. Occupational status: 9.5% unemployed seeking first or new job, 52.7% not in labor force, 37.9% full-time or part-time employee.

Sample comparison according to occupational status

A test of the independence assumption in the contingency table crossing occupational status (unemployed seeking first or new job, not in labor force, full-time or part-time employee) and sample (political news vs COVID-19 news administrations) produced a Bayes Factor (function contingencyTableBF() in the package BayesFactor) equal to 0.04, thus providing no evidence for the alternative hypothesis of association between the two variables.

Sample comparison according to marital status

A test of the independence assumption in the contingency table crossing marital status (married, cohabitation, not married/no cohabitation, divorced, widows/widowers) and sample (political news vs COVID-19 news administrations) produced a Bayes Factor equal to 0.01, thus providing no evidence for the alternative hypothesis of association between the two variables.

Data analysis

Data cleaning, manipulation, and analyses were performed in R program version 4.1.0 [41]. Descriptive analyses were facilitated by tidyverse package [42]. Bayesian modeling to assess the associations between the truth discernment and overall belief and the available covariates was performed in Rstudio using Stan and associated packages. Bayesian regression models were fitted using the brms package [43]. Markov chain Monte Carlo diagnostics for the final model were performed with (a) the potential scale reduction statistic ($\hat{R}$), (b) the ratio of the effective sample size to the total sample size drawn from the posterior distribution, and (c) trace plots of Markov chain Monte Carlo generated through the bayesplot package. Residual check and posterior predictive checks were also performed using the bayesplot package. The highest density interval of the posterior distribution was estimated using the bayestestR package. Prior to analysis, all continuous regression inputs were standardized to have a mean of 0 and a standard deviation of 1. As the values for predictors and the response were standardized, a weakly informative normal (0, 1) prior was used for the predictor slopes and intercept. Similarly, a weakly informative Cauchy (0, 1) prior was used for the model standard deviation (sigma).

Sensitivity

Truth discernment is akin to the sensitivity measure (d') of the Signal Detection Theory (SDT; [44]). In our sample, we found a correlation of 0.98 between truth discernment and d' when computing the d' index by using a multilevel Bayesian ordinal regression to fit a hierarchical unequal variance SDT model to the participants' ratings of the news headlines, with clustering (i.e., random effects) on the levels of participants and items.

Media consumption habits

Pairwise correlations between media consumption habits and spiritual practices, education level, age, and political liberal orientation are shown in Figure S1.

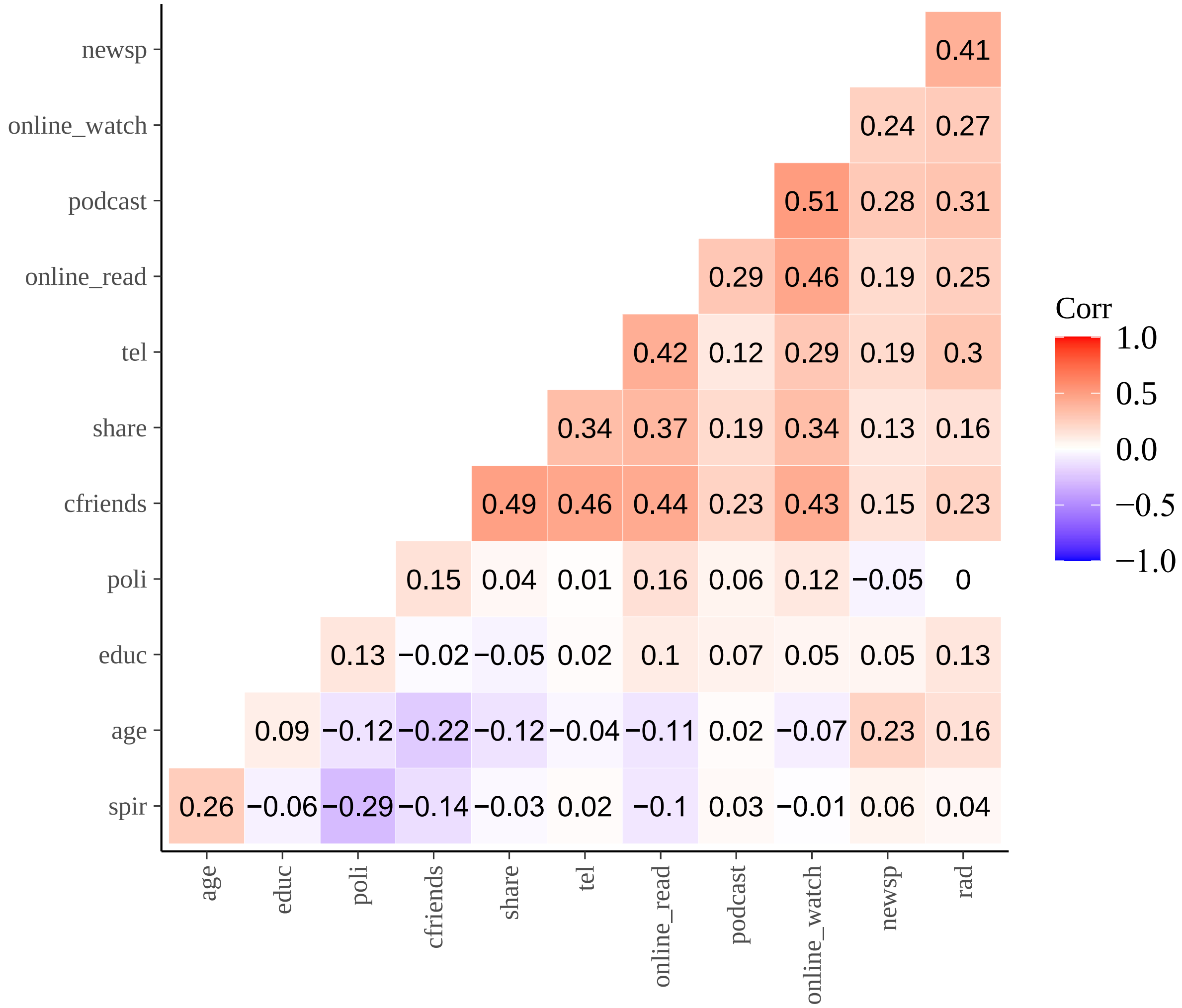

Figure S1. Pairwise correlations between media consumption habits and spiritual practices, education level, age, and political liberal orientation. newsp = reading newspapers, rad = listening to the news on the radio, tel = watching news

programs on television, online_read = reading news online, podcast = listening to news podcasts, online_watch = watching online news videos, cfriends= commenting news with friends, share = sharing news onsocial media.

According to [10], it is important to study the associations between news consumption and susceptibility to falling for fake news. Here, we examined whether the associations between truth discernment and overall belief (as dependent variables) and media consumption habits (as predictors) are modulated by the personal relevance of the news topics. To do so, we run a multivariate Bayesian regression analysis. In the present study, media consumption habits were measured by 8 items describing the weekly time spent by (1) reading newspapers, (2) listening to the news on the radio, (3) watching news programs on television, (4) reading news online, (5) listening to news podcasts, (6) watching online news videos, (7) commenting news with friends, and (8) sharing news on social media. The news category (political news versus COVID-19 news) was coded as a dummy variable interacting with the eight variables listed before. The comparison between the model including the interaction terms and the model without interactions was carried out via leave-one-out cross validation. The difference between the two models (ELPD-LOO difference = -6.7) was modest and not substantial (*SE* = 4.3). This means that the present data do not support the idea of different media consumption habits, for news with high or low personal relevance, when considering news truth discernment and credibility.

In both samples, *truth discernment* improved with the time spent watching news programs on television, β = 0.12, 95% CI [0.06, 0.18] and with the time spent reading news online, β = 0.14, 95% CI [0.07, 0.20], but decreased with the time spent sharing news on social media, β = -0.09, 95% CI [-0.15,-0.02]; when comparing the two samples, we found that truth discernment was higher for the political headline news than for the COVID-19 news, β = 0.66, 95% CI [0.55, 0.77].

In both samples, *overall belief* was higher for political news than for COVID-19 news, β = 0.13, 95% CI [0.02, 0.25], and increased with the time spent watching news programs on television, β = 0.10, 95% CI

[0.04, 0.17] and with the time spent reading news online, β = 0.07, 95% CI [0.00, 0.14], but decreased with the time spent watching online news videos, β = -0.09, 95% CI [-0.17, -0.02].

In summary, we found that familiarity (as measured by the time spent watching news programs on television and reading news online) not only increased participants' perception of news credibility [45], but also improved FN detection [7]. Interestingly, however, those participants who were less able to discern FN from TN were also those who spent more time sharing news on social media [46]. Participants attributed a lower credibility to COVID-19 news than to the political news, perhaps due to their lower domain knowledge in this field [47]. Our data thus confirm previous results concerning the relation between media habits, news credibility, and truth discernment.

Model comparison

In models' comparison, when the difference in ELPD-LOO is larger than 4, the number of observations is larger than 100, and the models are not badly misspecified, then $ELPD_{diff}$ $\pm 2SE$ can be interpreted as ~ *95*% credibility interval for the difference in LOO values between two models [48].

In the reference model, our purpose was not to include all the variables that had been found to affect FN vulnerability (the list would be too long), but rather to select those dimensions that, possibly, can differently affect participants' performance in the case of low or high personal relevance news topics. We excluded cognitive reflection, as indexed by the Cognitive Reflection Test (CRT), because this dimension (which role in FN vulnerability is well established; [8]) can be understood as a metacognitive trait with a high stability over time – *e.g.*, [49] reported a r = 0.755 for time points separated by over 2 years – this suggests that CRT it is quite invariant with respect to situational variables (including, possibly, the personal relevance of news topics). Measures of partisanship, conversely, appear to be less stable over time. For example, [50] argued that the temporal stability of right-wing authoritarianism may be weak, since this dimension can be considered to be more exogenous to a wide range of political attitudes, so that its temporal variance may in part reflect changes in the political environment or political rhetoric. Also other dimensions of FN vulnerability are less stable over time than CRT. Delusion proneness (as measured by the MIS), for example, shows a 2-years test-retest reliability of only r = 0.41 [51].

External validation

1. For the reference model based on the political news data, we fitted a Bayesian multiple regression model to predict either truth discernment or overall belief from 14 candidate predictors: gender ([52]; gender was coded as +0.5 = male, -0.5 = female), age [53], education [47], frequency of religious/spiritual behaviors [54], political orientation ([2]; political orientation was coded as follows: 1 = extreme right-wing affinity, ..., 8 = extreme left-wing affinity), two RWA sub-scales (aggression-submission, conventionalism; [55]), MIS [6], two RFS sub-scales [RFS positive, RFS negative; [6]), four FNSS sub-scales (paranormal beliefs, critical news consumption, conspiratorial beliefs, self-confirmatory bias; [19]).

2. After fitting the reference model, we used predictive projection to find the smallest possible submodel that would predict belief in FN (truth discernment or overall belief) almost as well as the reference model. For covariates selection, we used the predictive projection technique [56] as implemented in the package . The covariates were entered into the sub-models using a forward stepwise addition and the submodels' predictive performance was evaluated using the Expected Log Predictive Density (ELPD) obtained through the PSIS-LOO. For the optimal sub-model, we chose the smallest sub-model having an ELPD within 1 standard error of the reference model.

External validation for news truth discernment

For the political news data, we fitted a Bayesian multiple regression reference model to predict news truth discernment from 14 candidate predictors. For such model, the 95% CI did not cross the zero for eight covariates: age, β = 0.10, 95% CI [0.02, 0.18], gender, β = 0.29, 95% CI [0.12, 0.45], education, β = 0.12, 95% CI [0.04, 0.20], political orientation, β = 0.23, 95% CI [0.14, 0.31], Conventionalism, β = -0.26, 95% CI [-0.42, -0.09], MIS, β = -0.16, 95% CI [-0.27, -0.05], FNSS-Conspiratorial beliefs, β = -0.25, 95% CI [-0.34, -0.15], FNSS-Critical news consumption, β = 0.13, 95% CI [0.05, 0.21], FNSS-Self confirmation bias, β = -0.09, 95% CI [-0.17, -0.01]. Note that this list of explanatory variables does not coincide with the set of predictors selected by predictive projection in the next step of the analysis.

Out-of-sample predictive accuracy

Models' out-of-sample predictive accuracy was assessed using the LOO Bayesian estimate of the expected log pointwise predictive density (ELPD-LOO). The higher a model's ELPD-LOO score, the better that model's out-of-sample predictive accuracy. We used the Pareto k diagnostic in the loo package [57] to assess reliability of predictive fit. All considered models provided reasonable fits to the data ($k < 0.70$). When comparing models, we used the criterion $|\mathrm{ELPD_{diff}}/\mathrm{SE_{diff}}| \geq 2$ in order to considered the result noteworthy (Gelman et al., 2020). To evaluate how much variance a model explained in the data, we calculated the Bayesian $R^2 = \frac{\mathrm{Var_{fit}}}{\mathrm{Var_{fit}}+\mathrm{Var_{res}}}$, which is a generalization of the traditional R^2, but has the advantage of incorporating posterior uncertainty [58]. Bayesian R^2 and ELPD-LOO can make different patterns of predictions because ELPD-LOO is a measure of the ability of the model to predict new observations, not a measure of how well a model explains the sample data.

Evaluation of models' performance

Models' performance was evaluated according to several criteria. (a) To perform external validation according to Scenario 1, we first we computed the log-likelihood for each observation in the political news data. When fitting the model in the COVID-19 news data, we recomputed the log-likelihood for each observation using the posterior draws conditional on the training data, but now we passed in the COVID-19 news data to get the log predictive densities for the COVID-19 news data. (b) To perform external validation according to Scenario 2, we carried out model comparison by considering the difference in the models' expected predictive accuracy as estimated by their difference in ELPD-LOO.

Predictive projection

The problem of finding the best predictors subset in the context of multiple regression has been traditionally dealt with by using statistical techniques such as stepwise selection (which is based on p-values criterion), or information criteria (such as AIC or BIC), or machine learning methods (such as LASSO, in which the predictor slopes are continuously increased towards their least-squares solution). All the above-mentioned methods, however, suffer from the problem of overfitting. Because of overfitting, models obtained with such techniques tend to provide a very good explanations of the data at hand (e.g., high R^2, low RMSE), but their performance does not generalize and they perform poorly with new sets of data. In the case of such methods, moreover, p-values and confidence intervals are invalid because they are computed by ignoring the selection procedure.

The problem of finding the best predictors subset has recently found a new solution within a Bayesian framework in terms of the predictive projection feature selection method [56]. At the first step, a reference model is fitted using all available predictors. At the second step, smaller submodels are fitted to approximate the reference model's predictions, using projection. The covariates in the best model for each submodel size are identified by decreasing the Kullback–Leibler divergence from the reference model to the projected submodel using a forward stepwise addition. The procedure selects the submodel with the smallest number of predictors which has a similar predictive power as the reference model, judged by the mean log predictive density and by the root mean square error. To avoid overfitting, at a third step, the selected submodel is compared to the reference model on cross-validated prediction accuracy by using Pareto-smoothed importance sampling leave-one-out cross-validation (PSIS-LOO). Differently from other information criteria, the posterior uncertainty is taken into account when estimating predictive accuracy; thus LOO provides a more accurate measure of model fit.

Reference model for political news truth discernment

Posterior mean, standard error, 95% credible interval and $\hat{R}$ statistic for each parameter of the model predicting political news truth discernment are shown in Table S1. The legend of Table S1 is reported below.

Table S1. Posterior mean, standard error, 95% credible interval and $\hat{R}$ statistic for each parameter of the model predicting political news truth discernment.

Parameter	Mean	SE	Lower Bound	Upper Bound	Rhat
Intercept	0.687	0.108	0.474	0.898	1.000
age	0.102	0.042	0.019	0.184	1.000
sex	0.288	0.084	0.123	0.453	1.000
educ	0.122	0.042	0.041	0.204	1.000
poli	0.228	0.044	0.141	0.314	1.000
spir	0.082	0.058	-0.031	0.194	1.000
agsu	-0.028	0.091	-0.206	0.153	1.000
conv	-0.255	0.085	-0.422	-0.090	1.000
miis	-0.161	0.057	-0.272	-0.048	1.000
rfsp	-0.005	0.066	-0.136	0.124	1.000
rfsn	0.033	0.059	-0.082	0.149	1.000
para	0.031	0.046	-0.059	0.121	1.000
cosp	-0.248	0.048	-0.342	-0.155	1.000
crit	0.132	0.041	0.051	0.212	1.000

Parameter	Mean	SE	Lower Bound	Upper Bound	Rhat
conf	-0.091	0.041	-0.172	-0.012	1.000
sigma	0.810	0.027	0.759	0.866	1.000

Legend: *educ = Education level (specified as number of years of education completed); poli = Liberal orientation (participants indicated how politically liberal or conservative they were on a scale from 1-extremely conservative to 8-extremely liberal in terms of their general outlook, social policy, and economic policy [59]; higher scores indicated greater political liberalism; spir = Religious/spiritual practices and behaviors, such as prayer, meditation, or participating in collective practices, such as attending a place of worship (specified as number of hours per week); agsu = Right-Wing Authoritarianism Scale (RWAS) - Aggression-submission; conv = Right-Wing Authoritarianism Scale (RWAS) - Conservatorism; miis = Magical Ideation Scale (MIS); rfsp = Religious Fundamentalism Scale (RFS) - Belief; rfsn = Religious Fundamentalism Scale (RFS) - Skepticism; para = Fake News Susceptibility Scale (FNSS) - Irrational beliefs; cosp = Fake News Susceptibility Scale (FNSS) - Conspiratorial beliefs; crit = Fake News Susceptibility Scale (FNSS) - Critical news consumption; conf = Fake News Susceptibility Scale (FNSS) - Self-beliefs.*

Reference model for political news overall belief

Posterior mean, standard error, 95% credible interval and $\hat{R}$ statistic for each parameter of the model predicting political news truth discernment are shown in Table S2. The legend of Table S2 is reported below.

Table S2. Posterior mean, standard error, 95% credible interval and $\hat{R}$ statistic for each parameter of the model predicting political news truth discernment.

Parameter	Mean	SE	Lower Bound	Upper Bound	Rhat
Intercept	0.134	0.136	-0.131	0.403	1.000
age	0.104	0.053	0.000	0.207	1.000
sex	0.015	0.105	-0.190	0.222	1.000
educ	0.143	0.052	0.042	0.245	1.000
poli	-0.032	0.055	-0.141	0.076	1.000
spir	-0.064	0.072	-0.206	0.078	1.000
agsu	0.137	0.113	-0.085	0.361	1.000
conv	-0.012	0.106	-0.221	0.197	1.000
miis	0.151	0.072	0.013	0.291	1.000
rfsp	0.043	0.083	-0.122	0.207	1.000
rfsn	-0.010	0.074	-0.155	0.134	1.000
para	0.058	0.058	-0.056	0.172	1.000
cosp	-0.115	0.060	-0.232	0.001	1.000
crit	0.074	0.052	-0.028	0.176	1.000

Parameter	Mean	SE	Lower Bound	Upper Bound	Rhat
conf	0.226	0.051	0.126	0.325	1.000
sigma	1.012	0.034	0.948	1.082	1.000

Legend: *educ = Education level (specified as number of years of education completed); poli = Liberal orientation (participants indicated how politically liberal or conservative they were on a scale from 1-extremely conservative to 8-extremely liberal in terms of their general outlook, social policy, and economic policy [59]; higher scores indicated greater political liberalism; spir = Religious/spiritual practices and behaviors, such as prayer, meditation, or participating in collective practices, such as attending a place of worship (specified as number of hours per week); agsu = Right-Wing Authoritarianism Scale (RWAS) - Aggression-submission; conv = Right-Wing Authoritarianism Scale (RWAS) - Conservatorism; miis = Magical Ideation Scale (MIS); rfsp = Religious Fundamentalism Scale (RFS) - Belief; rfsn = Religious Fundamentalism Scale (RFS) - Skepticism; para = Fake News Susceptibility Scale (FNSS) - Irrational beliefs; cosp = Fake News Susceptibility Scale (FNSS) - Conspiratorial beliefs; crit = Fake News Susceptibility Scale (FNSS) - Critical news consumption; conf = Fake News Susceptibility Scale (FNSS) - Self-beliefs.*

Reference model for COVID-19 news truth discernment

Posterior mean, standard error, 95% credible interval and $\hat{R}$ statistic for each parameter of the model predicting political news truth discernment are shown in Table S3. The legend of Table S3 is reported below.

Table S3. Posterior mean, standard error, 95% credible interval and $\hat{R}$ statistic for each parameter of the model predicting political news truth discernment.

Parameter	Mean	SE	Lower Bound	Upper Bound	Rhat
Intercept	-0.337	0.052	-0.439	-0.235	1.000
age	0.157	0.036	0.087	0.228	1.000
sex	0.162	0.069	0.026	0.297	1.000
educ	0.093	0.030	0.033	0.153	1.000
poli	0.064	0.035	-0.004	0.132	1.000
spir	0.099	0.046	0.009	0.190	1.000
agsu	-0.014	0.050	-0.113	0.083	1.000
conv	-0.132	0.055	-0.239	-0.025	1.000
miis	-0.162	0.033	-0.227	-0.097	1.000
rfsp	-0.009	0.049	-0.104	0.086	1.000
rfsn	-0.032	0.044	-0.118	0.054	1.000
para	-0.095	0.038	-0.170	-0.022	1.000
cosp	-0.231	0.036	-0.303	-0.159	1.000
crit	0.173	0.030	0.115	0.231	1.000

Parameter	Mean	SE	Lower Bound	Upper Bound	Rhat
conf	0.042	0.030	-0.018	0.101	1.000
sigma	0.747	0.021	0.706	0.790	1.000

Legend: *educ = Education level (specified as number of years of education completed); poli = Liberal orientation (participants indicated how politically liberal or conservative they were on a scale from 1-extremely conservative to 8-extremely liberal in terms of their general outlook, social policy, and economic policy [59]; higher scores indicated greater political liberalism; spir = Religious/spiritual practices and behaviors, such as prayer, meditation, or participating in collective practices, such as attending a place of worship (specified as number of hours per week); agsu = Right-Wing Authoritarianism Scale (RWAS) - Aggression-submission; conv = Right-Wing Authoritarianism Scale (RWAS) - Conservatorism; miis = Magical Ideation Scale (MIS); rfsp = Religious Fundamentalism Scale (RFS) - Belief; rfsn = Religious Fundamentalism Scale (RFS) - Skepticism; para = Fake News Susceptibility Scale (FNSS) - Irrational beliefs; cosp = Fake News Susceptibility Scale (FNSS) - Conspiratorial beliefs; crit = Fake News Susceptibility Scale (FNSS) - Critical news consumption; conf = Fake News Susceptibility Scale (FNSS) - Self-beliefs.*

Reference model for COVID-19 news overall belief

Posterior mean, standard error, 95% credible interval and $\hat{R}$ statistic for each parameter of the model predicting political news truth discernment are shown in Table S4. The legend of Table S4 is reported below.

Table S4. Posterior mean, standard error, 95% credible interval and $\hat{R}$ statistic for each parameter of the model predicting political news truth discernment.

Parameter	Mean	SE	Lower Bound	Upper Bound	Rhat
Intercept	-0.158	0.065	-0.286	-0.031	1.000
age	-0.081	0.045	-0.169	0.006	1.000
sex	-0.183	0.087	-0.355	-0.011	1.000
educ	0.020	0.038	-0.055	0.094	1.000
poli	-0.007	0.043	-0.091	0.078	1.000
spir	-0.111	0.058	-0.225	0.002	1.000
agsu	-0.135	0.062	-0.256	-0.015	1.000
conv	-0.230	0.068	-0.364	-0.097	1.000
miis	-0.027	0.041	-0.108	0.054	1.000
rfsp	0.145	0.061	0.025	0.264	1.000
rfsn	-0.073	0.055	-0.180	0.035	1.000
para	0.129	0.047	0.037	0.221	1.000
cosp	0.022	0.045	-0.067	0.111	1.000
crit	-0.029	0.037	-0.103	0.044	1.000

Parameter	Mean	SE	Lower Bound	Upper Bound	Rhat
conf	0.122	0.038	0.048	0.196	1.000
sigma	0.936	0.026	0.886	0.990	1.000

Legend: *educ = Education level (specified as number of years of education completed); poli = Liberal orientation (participants indicated how politically liberal or conservative they were on a scale from 1-extremely conservative to 8-extremely liberal in terms of their general outlook, social policy, and economic policy [59]; higher scores indicated greater political liberalism; spir = Religious/spiritual practices and behaviors, such as prayer, meditation, or participating in collective practices, such as attending a place of worship (specified as number of hours per week); agsu = Right-Wing Authoritarianism Scale (RWAS) - Aggression-submission; conv = Right-Wing Authoritarianism Scale (RWAS) - Conservatorism; miis = Magical Ideation Scale (MIS); rfsp = Religious Fundamentalism Scale (RFS) - Belief; rfsn = Religious Fundamentalism Scale (RFS) - Skepticism; para = Fake News Susceptibility Scale (FNSS) - Irrational beliefs; cosp = Fake News Susceptibility Scale (FNSS) - Conspiratorial beliefs; crit = Fake News Susceptibility Scale (FNSS) - Critical news consumption; conf = Fake News Susceptibility Scale (FNSS) - Self-beliefs.*

Data and R scripts

We have made the data and the R scripts necessary to run the present analyses available online as a snakemake workflow (https://anonymous.4open.science/r/fakenews-anonymous-2CC8/README.md).

References

1. Shu, K.; Sliva, A.; Wang, S.; Tang, J.; Liu, H. Fake news detection on social media: A data mining perspective. *Explor. Newsl.* **2017**, *19*, 22–36.
2. Lazer, D.M.; Baum, M.A.; Benkler, Y.; Berinsky, A.J.; Greenhill, K.M.; Menczer, F.; Zittrain, J.L. The science of fake news. *Science* **2018**, *359*, 1094–1096.
6. Bronstein, M.V.; Pennycook, G.; Bear, A.; Rand, D.G.; Cannon, T.D. Belief in fake news is associated with delusionality, dogmatism, religious fundamentalism, and reduced analytic thinking., *J. Appl. Res. Mem. Cogn.* **2019**, *8*, 108–117.
7. Pennycook, G.; Rand, D.G. Who falls for fake news? The roles of bullshit receptivity, overclaiming, familiarity, and analytic thinking. *J. Pers.* **2020**, *88*, 185–200.
8. Pennycook, G.; Rand, D.G. Lazy, not biased: Susceptibility to partisan fake news is better explained by lack of reasoning than by motivated reasoning. *Cognition* **2019**, *188*, 39–50.
10. Sindermann, C.; Cooper, A.; Montag, C. A short review on susceptibility to falling for fake political news. *Curr. Opin. Psychol.* **2020**, *36*, 44–48.
18. Rubin, V.L.; Conroy, N.; Chen, Y.; Cornwell, S. Fake news or truth? Using satirical cues to detect potentially misleading news. In Proceedings of the Second Workshop on Computational Approaches to Deception Detection, San Diego, CA, USA, 17 June 2016; pp. 7–17.
19. Giuntoli, L.; Capuozzo, P.; Ceccarini, F.; Colpizzi, I.; Caudek, C. Development and validation of the Fake News Supsceptibility Scale.
20. Rattazzi, A.M.M.; Bobbio, A.; Canova, L. A short version of the Right-Wing Authoritarianism (RWA) Scale. *Pers. Individ. Differ.* **2007**, *43*, 1223–1234.
21. Sinclair, A.H.; Stanley, M.L.; Seli, P. Closed-minded cognition: Right-wing authoritarianism is negatively related to belief updating following prediction error. *Psychon. Bull. Rev.* **2020**, *27*, 1348–1361.
22. Eckblad, M.; Chapman, L.J. Magical ideation as an indicator of schizotypy. *J. Consult. Clin. Psychol*. **1983**, *51*, 215–225.
23. Martin, J.G.; Westie, F.R. The tolerant personality. *Am. Sociol. Rev.* **1959**, *24*, 521–528.
35. Vlachos, A.; Riedel, S. Fact checking: Task definition and dataset construction. *Proc. ACL 2014 Workshop Lang. Technol. Comput. Soc. Sci.* **2014**, 18–22.
36. Klayman, J. Varieties of confirmation bias. *Psychol. Learn. Motiv.* **1995**, *32*, 385–418.
37. Altemeyer, B. The other "authoritarian personality". *Adv. Exp. Soc. Psychol.* **1998**, *30*, 47–92.
38. Garzitto, M.; Picardi, A.; Fornasari, L.; Gigantesco, A.; Sala, M.; Fagnani, C.; Stazi, M.A.; Ciappolino, V.; Fabbro, F.; Altamura; A.C.; et al. Normative data of the Magical Ideation Scale from childhood to adulthood in an Italian cohort. *Compr. Psychiatry* **2016**, *69*, 78–87.
39. Chapman, L.J.; Chapman, J.P. The search for symptoms predictive of schizophrenia. *Schizophr. Bull.* **1987**, *13*, 497–503.
40. Carlucci, L.; Tommasi, M.; Saggino, A. Factor structure of the Italian version of the religious fundamentalism scale. *Psychol. Rep.* **2013**, *112*, 6–13.
41. R Core Team. R: A language and environment for statistical computing. R Foundation for Statistical Computing: Vienna, Austria, 2021. Available online: https://www.R-project.org/.
42. Wickham, H.; Averick, M.; Bryan, J.; Chang, W.; McGowan, L.D.; François, R.; Grolemund, G.; Hayes, A.; Henry, L.; Hester, J.; et al. Welcome to the tidyverse. *J. Open Source Softw.* **2019**, *4*, 1686.
43. Bürkner, P.-C. Advanced Bayesian multilevel modeling with the R package brms. *The R Journal* **2018**, *10*, 395–411.
44. Green, D.M.; Swets, J.A. *Signal Detection Theory and Psychophysics*. Wiley: New York, NY, USA, 1966.
45. Metzger, M.J.; Hartsell, E.H.; Flanagin, A.J. Cognitive dissonance or credibility? A comparison of two theoretical explanations for selective exposure to partisan news. *Commun. Res.* **2020**, *47*, 3–28.
46. Ross, R.M.; Rand, D.G.; Pennycook, G. Beyond" fake news": Analytic thinking and the detection of false and hyperpartisan news headlines. *Judgm. Decis. Mak.* **2021**, *16*, 484–504.
47. Zrnec, A.; Poženel, M.; Lavbič, D. Users' ability to perceive misinformation: An information quality assessment approach. *Inf. Process. Manag.* **2022**, *59*, 102739.
48. Gelman, A.; Goodrich, B.; Gabry, J.; Vehtari, A. R-squared for bayesian regression models. *Am. Stat.* **2019**, 307–309.
49. Stagnaro, M.; Pennycook, G.; Rand, D.G. Performance on the cognitive reflection test is stable across time. *Judgm. Decis. Mak.* **2018**, *13*, 260–267.
50. Engelhardt, A.M.; Feldman, S.; Hetherington, M.J. Advancing the measurement of authoritarianism. *Political Behav.* **2021**, 1–24.

51. Meyer, T.D.; Hautzinger, M. Two-year stability of psychosis proneness scales and their relations to personality disorder traits. *J. Personal. Assess.* **1999**, *73*, 472–488.
52. Lai, K.; Xiong, X.; Jiang, X.; Sun, M.; He, L. Who falls for rumor? Influence of personality traits on false rumor belief. *Personal. Individ. Differ.* **2020**, *152*, 109520.
53. Brashier, N.M.; Schacter, D.L. Aging in an era of fake news. *Curr. Dir. Psychol. Sci.* **2020**, *29*, 316–323.
54. Pennycook, G.; Cheyne, J.A.; Koehler, D.J.; Fugelsang, J.A. On the belief that beliefs should change according to evidence: Implications for conspiratorial, moral, paranormal, political, religious, and science beliefs. *Judgm. Decis. Mak.* **2020**, *15*, 476–498.
55. Alper, S.; Bayrak, F.; Yilmaz, O. Psychological correlates of COVID-19 conspiracy beliefs and preventive measures: Evidence from turkey. *Curr. Psychol.* **2020**, 1–10.
56. Piironen, J.; Paasiniemi, M.; Vehtari, A. Projective inference in high-dimensional problems: Prediction and feature selection. *Electron. J. Stat.* **2020**, *14*, 2155–2197.
57. Vehtari, A.; Gelman, A.; Gabry, J. Practical bayesian model evaluation using leave-one-out cross-validation and WAIC. *Stat. Comput.* **2017**, *27*, 1413–1432.
58. Gelman, A.; Hill, J.; Vehtari, A. *Regression and other stories*. Cambridge University Press: Cambridge, UK, 2020.
59. Skitka, L.J.; Mullen, E.; Griffin, T.; Hutchinson, S.; Chamberlin, B. Dispositions, scripts, or motivated correction? Understanding ideological differences in explanations for social problems. *J. Personal. Soc. Psychol.* **2002**, *83*, 470–487.
